# Persona preparedness: a survey instrument for measuring the organizational readiness for deploying personas

**DOI:** 10.1007/s10799-022-00373-9

**Published:** 2022-09-13

**Authors:** Joni Salminen, Lene Nielsen, Malik Bahloul, Rasmus Grønlund Jørgensen, João M. Santos, Soon-Gyo Jung, Bernard J. Jansen

**Affiliations:** 1 Qatar Computing Research Institute, Hamad Bin Khalifa University, HBKU Research Complex, RC1, Doha, Qatar; 2 Turku School of Economics, University of Turku, Turku, Finland; 3 School of Marketing and Communication, University of Vaasa, Vassa, Finland; 4 IT University of Copenhagen, Copenhagen, Denmark; 5 Instituto Universitário de Lisboa (ISCTE-IUL), Lisbon, Portugal; 6 Department of Human-AI Interaction, Sungkyunkwan University, Seoul, South Korea

**Keywords:** User empathy, Survey instrument, Personas, Human-centered IT, Human–computer interaction

## Abstract

User-centric design within organizations is crucial for developing information technology that offers optimal usability and user experience. Personas are a central user-centered design technique that puts people before technology and helps decision makers understand the needs and wants of the end-user segments of their products, systems, and services. However, it is not clear how ready organizations are to adopt persona thinking. To address these concerns, we develop and validate the Persona Readiness Scale (PRS), a survey instrument to measure organizational readiness for personas. After a 12-person qualitative pilot study, the PRS was administered to 372 professionals across different industries to examine its reliability and validity, including 125 for exploratory factor analysis and 247 for confirmatory factor analysis. The confirmatory factor analysis indicated a good fit with five dimensions: Culture readiness, Knowledge readiness, Data and systems readiness, Capability readiness, and Goal readiness. Higher persona readiness is positively associated with the respondents’ evaluations of successful persona projects. Organizations can apply the resulting 18-item scale to identify areas of improvement before initiating costly persona projects towards the overarching goal of user-centric product development. Located at the cross-section of information systems and human–computer interaction, our research provides a valuable instrument for organizations wanting to leverage personas towards more user-centric and empathetic decision making about users.

**Supplementary Information:**

The online version contains supplementary material available at 10.1007/s10799-022-00373-9.

## Introduction

User-centric decision making is seen as impactful for creating products, services, and information technology that better serves end-user needs by offering optimal usability and user experience (UX) [29, 60, 87, 113]. Human–computer interaction (HCI) researchers have introduced multiple user-centered design techniques that aim to improve organizations’ ability to develop solutions that are user friendly and offer a high-quality UX [22, 107]. One of these techniques is personas, originating from HCI in the late 1990s and later spreading to information systems (IS), marketing/business, and other domains that deal with human-centered decision making to improve the usability and UX of systems and products [34, 63, 73, 112]. Personas are fictitious user types [26] that represent the needs, wants, and circumstances of central end-user or customer groups that use or are intended to use a given system, technology, product, or service [46, 55, 75]. Personas are applied in design, communication, software development, marketing, and other processes requiring user-centered thinking [56, 84].

**Fig. 1:**
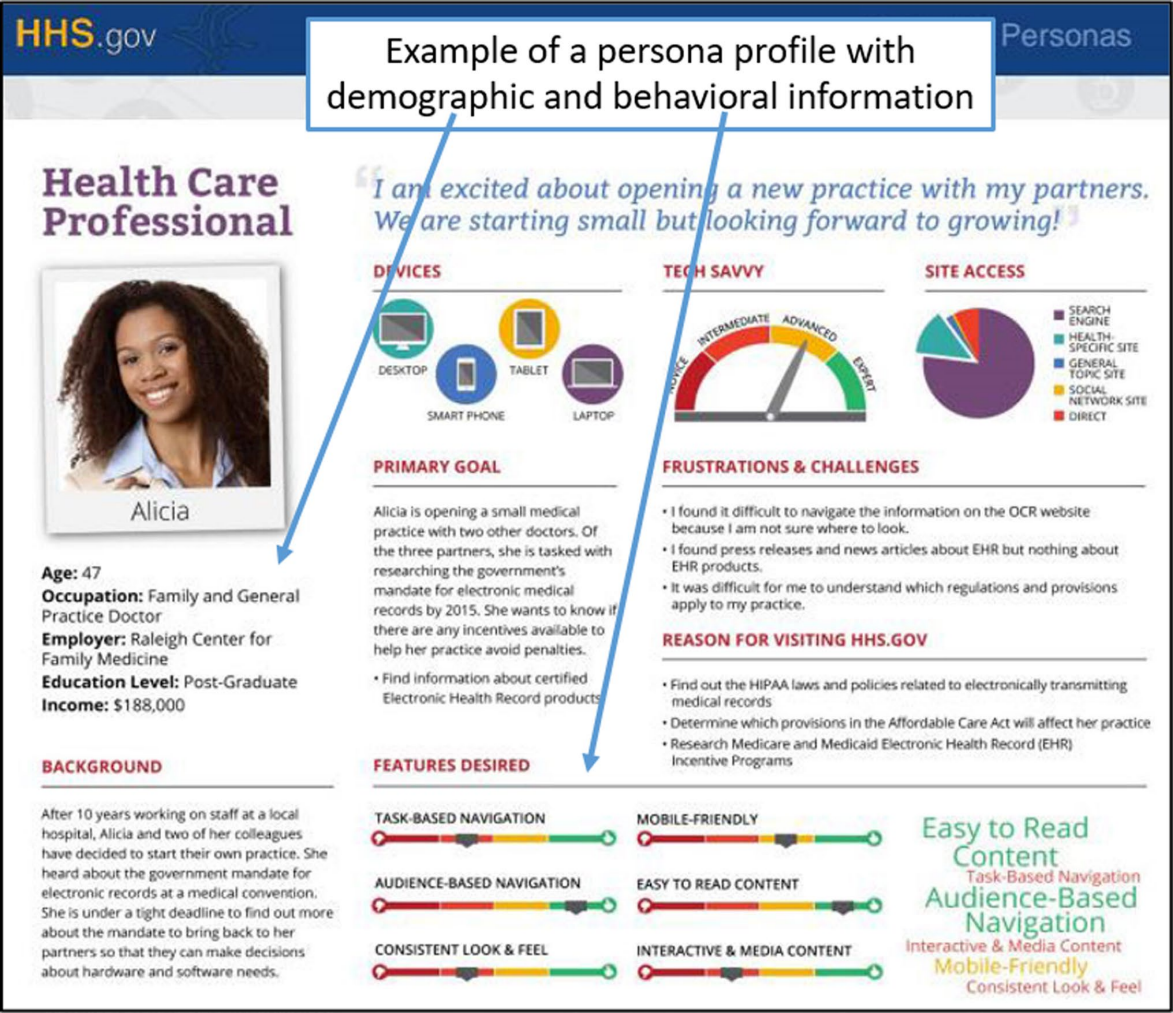
Example of a persona profile (Source:https://s3.amazonaws.com/digitalgov/_legacy-img/2014/12/765-x-570-Complex-Persona.jpg)

Studies report various benefits associated with personas, such as aligning user understanding and communication within a design team [38, 50], increasing the level of empathy or user-centricity [30, 40, 81], and avoiding self-centered bias in product design and development activities [26, 37]. There is also evidence of persona projects yielding a financially positive return on investment [108], increasing marketing performance [97], and promoting inclusivity and user well-being. Due to their flexible nature, personas can be rapidly deployed for understanding user behaviors during turbulent times, such as the COVID-19 pandemic and other circumstances requiring a rapid understanding of various human segments. An example of a persona profile is shown in Fig. 1.

Research shows that researchers and practitioners consistently display interest in creating and using personas towards user-centric design goals [36, 75, 90], but they nonetheless struggle to implement personas in active day-to-day use [3, 35, 70]. The adoption and active use of personas are hindered by factors such as perceived lack of credibility, accuracy, or usefulness [35, 42, 70, 89, 92]. While prior research has focused on persona perceptions [100] as the explanation for why personas fail, the importance of organizational factors concerning successful persona implementation is often overlooked. Yet, organizational factors tend to play a central role in creating better information systems and products [12, 12, 21, 59]. A crucial observation in this regard is that IS and HCI have “shared concerns” [116] (p. 397) in terms of developing systems that have real-world value and impact – yet, these two disciplines often fail to share literature, theory, and findings; an effort that would benefit both fields [116].

Therefore, in this study, we take the viewpoint that many of the observed challenges with persona projects (an HCI perspective to enhance user-centric thinking and resultant usability and UX) can be attributed to organizations’ lack of *readiness* for implementing personas (an IS perspective that systems are incorporated in real organizations and adopted by teams with multiple constraints and predispositions). In other words, despite the best intentions of persona creators and the target organization’s willingness to create great products, personas often fail in reality because the organizations lack the required antecedents (“readiness factors”) for successful persona implementation.

As such, the concept of persona readiness addresses the question: “Are we, as an organization, fully equipped to implement personas?” According to our experience of witnessing multiple persona projects, this question is rarely asked before creating and deploying personas, which may partially explain the reported alarming failure cases in organizations. More specifically, the lack of readiness may be associated with the broader organizational scheme of things, including factors such as awareness, culture, skills, and capabilities, and lack of articulation of goals and metrics for persona projects [35, 42, 70, 89].

To remedy such matters, an organization interested in making personas work for them first needs to be aware of the specific issues. This *situational awareness* provides the organization with the necessary mindset to address specific issues to improve their persona readiness, which, in turn, is aimed at enhancing the success of the overall persona project. This is vital because organizations may not always be aware of what a successful persona project requires in the first place. For example, they may underestimate the effort required for training team members on how to actually use the personas or presume that simply having some customer data enables the creation of high-quality personas for decision making. Based on our experience in the field, spanning many years and multiple persona projects, such conflated expectations are common. For example, many organizations assume that since they have a social media account, they can generate data-driven personas, even though the extant methodologies typically impose specific requirements for the amount and structure of data [6, 51, 53]. This is not to say that people in organizations would be ignored because of a lack of interest—quite the opposite; they want to learn about personas. Nonetheless, the lack of knowledge hinders the success of persona projects within the organizations that employ these people.

Based on the above reasoning, this research addresses the crux of the matter; that there is currently no easy way to systematically gauge the organization’s current state and how well that state is compatible with the optimal environmental circumstances for a successful persona implementation. Towards this end, we develop and validate the *Persona Readiness Scale* (PRS), a survey instrument to evaluate how equipped organizations are for persona implementation. Our goal for creating the PRS is to make it easily deployable (i.e., not long and difficult to understand) for all types of organizations, while still capturing the essential dimensions of what makes an organization ready for personas. Developing this instrument brings about two key benefits for organizations:

- **Benefit 1:** The PRS serves persona advocates that need tools that help them introduce and diffuse personas more effectively in their organizations.
- **Benefit 2:** The PRS helps practitioners carry out evidence-based interventions that improve the organization’s readiness to initiate persona projects.

This study builds upon prior work [98]. The current study considerably expands that work, by adding a more in-depth literature review, providing a pilot study that modifies the items and also adds some new ones, collecting an extensive empirical sample of more than 300 organizations, as well as conducting a statistical analysis to validate the scale with an extensive sample of respondents. Thus, the validated scale substantially improves the first version based nearly solely on a review of the literature and not empirically used in the field, and it demonstrates why it is critically important to report the results and the process of obtaining the results. Overall, the scale can be of interest to scholars and practitioners working in various fields, including IS/HCI researchers, cognitive ergonomists, software and system designers, and strategic management.

## Theoretical background

Personas are imaginary people representing real users of unique user segments [26], and personas are an HCI technique that is one of the closest to incorporating human embodiment for design tasks, with the possible exception of direct user feedback [27]. Personas represent the goals, needs, and wants of a readily distinguishable audience, customer, or user groups [8, 46, 77] by presenting this information in a digestible format. Personas are applied in research and industry [2]. A longitudinal literature review of HCI research has shown that personas are continuously deployed and studied [36]. Personas are deployed in requirements systems engineering, development of products, UX/UI design, user support requirements, advertising, marketing, and other user or customer understanding fields [10, 23, 24, 37, 47, 84]. Personas are nearly always presented in profiles displaying various information fields, such as pictures, names, demographics, and the goals and wants of the persona [76, 91]. The overall aim of personas is to assist designers in empathizing with various users [9, 65]. Therefore, personas are key instruments for the user-centered design of products or services.

Criticism of personas is common in the literature, however. Often, the criticized aspects include the lack of methodological robustness, small sample sizes, lack of accuracy and precision, difficulty of evaluation, and unproven use cases and benefits [24, 35, 48, 70, 89, 93]. Roughly speaking, the points of critique can be categorized to persona creation, evaluation, and implementation [93]. While there are certainly challenges in all these areas [48], one of the key issues is that personas are often not correctly implemented in organizations. For instance, Rönkkö et al. (2004) report a case where applying personas to a software development project failed, specifically arguing that “*The problem was not with the user; socio-political factors in the branch in which the software was developed proved to be of much greater importance.*” (p. 112). Nielsen and Storgaard Hansen [77] explicitly mention lack of organizational maturity as a possible root cause for persona failure, whereas Seidelin et al. [103] present preliminary evidence of the association between persona success and UX maturity. In a user study by Billestrup et al. [17], one participant argues that lack of maturity was blocking the organization’s implementation of personas: “*I would like to introduce personas in my current employment but the company needs to be at a higher level of maturity before it would make sense*.” (p. 256). This quotation contains insightful thinking in that personas require certain prerequisites from the organization, which are often ignored.

Overall, these findings imply that organizational factors, such as participation, empowerment, and the development of routines influence the success of persona projects [89]. Consequently, demonstrating the real benefits of personas for an organization has proven to be difficult. Findings from empirical persona studies [35, 70, 78] support the notion that organizational factors are highly influential for persona projects' eventual success or failure. Therefore, two logical extensions follow: (a) *organizations, in some cases, may not possess the adequate readiness for taking on personas*, and (b) *organizations may vary by their readiness for personas*. To this end, reviewing persona studies,^1^ we devised a list of *possible* indicators that characterize the extreme cases of low and high persona readiness. ‘Possible’ means that these indicators are directly or indirectly implied but typically not empirically shown in previous studies.

More precisely, organizations with low persona readiness exhibit the following qualities:

- Do not perceive a need for personas. Do not consider personas important. Do not think personas would be useful.
- Do not think user understanding is crucial. Do not think empathy is needed for understanding users, defining requirements, and making product decisions.
- Do not understand the concept of personas. Do not have a clear picture of applying personas in real use cases.
- Do not have a “champion” for personas. Do not have a budget for persona creation and implementation. Do not provide training for team members about personas.
- Do not actively collect user data. Do not have much user data. The user data is dated. The user data is shallow.
- Do not have data science expertise. Do not have advanced user segmentation know-how.
- Do not have a plan for implementing personas after their creation. Do not have goals for persona use. Do not have clear use cases. Do not have defined quantitative metrics for goal attainment.
  In turn, organizations with high persona readiness exhibit the following qualities:
- Perceive a need for personas. Consider personas important. Think personas would be useful for them.
- Believe user understanding is crucial. Believe empathy is needed for understanding users, defining requirements, and making product decisions.
- Understand the concept of personas. Have a clear picture of applying personas in real use cases.
- Have a “champion” for personas. Have a budget for persona creation and implementation. Provide training for team members not familiar with personas.
- Actively collect user data. Have much user data, including behavioral and demographic information on the users. The user data is updated. The user data is rich, including user interviews or written feedback.
- Have data science expertise. Have advanced user segmentation know-how.
- Have a plan for implementing personas after their creation. Have quantitative goals for persona use. Have defined clear use cases. Have defined quantitative metrics for goal attainment.

These characteristic differences in organizations’ persona readiness can possibly explain the divergent views in the literature, with some authors arguing that personas are not applicable [89] and others arguing they are applicable [77]. If organizational readiness for personas indeed varies and affects the success of a project, it would be a grave mistake for an unready organization to engage in a persona project. This would reflect premature commitment and result in skepticism towards the method. In turn, if the organization is able to quantify its readiness, it can then systematically work towards improving its readiness along specific dimensions or indicators. This conceptual starting point is the offset for the development of the PRS, an instrument for measuring organizational readiness for persona implementation.

## Methodology

### Research strategy

An essential question that follows from the premises posed is *how to measure organizational readiness for personas?* Conceptually relevant constructs and items from other research domains can be adapted for this objective. Therefore, we need to first establish a conceptual understanding of the facets of persona readiness. We begin by investigating technology readiness and maturity scales from existing literature so as to identify constructs and items (i.e., statements, questions) that researchers have developed to measure the readiness/maturity of an organization to adopt user-centered technologies, such as big data, analytics, UX tools, or data science. Our premise is that the readiness for such technologies reflects the readiness for other user-centric design methods, such as personas.

The goal is to make the scale applicable to different persona types, including qualitative, quantitative, and mixed-method personas [48]. This means the scale needs to address different capabilities and skills for persona creation. For example, it needs to include indicators that assess the organization’s ability to work with data-driven personas [49, 72], which are a subtype of quantitative personas that rely on data science algorithms and online analytics data. This implies that the development of the scale can benefit from studies that have developed instruments for measuring technology readiness, analytics readiness, Big Data readiness, or artificial intelligence (AI) readiness.

Furthermore, readiness and maturity models regarding user experience (UX) and related applications [25, 33, 66, 102] can offer inspiration because “maturity” is conceptually similar to “readiness” [102]. The main difference in our understanding of readiness is that it offers an insight into the preparedness for *starting* with personas rather than the maturity of *using* personas. Therefore, it is necessary to develop a new instrument to specifically address aspects of readiness to implement personas, rather than the maturity of using them—but, in this process, dimensions and items from technological and UX maturity should be considered a source of inspiration. As personas contain specific considerations, existing maturity scales may not be directly applicable to the context of personas, and a new scale explicitly developed for personas is needed.

### Literature searches and screening

Conceptually, readiness has the connotation of being ready (or not) to start a persona project. In other words, the question is “Is your organization ready to start with personas?”. Naturally, the question could also be formulated as “Is your organization mature enough for personas?”, which implies a conceptual linkage with the various (technology) maturity models in HCI and IS research. Therefore, we included both concepts, readiness, and maturity in our literature searches to find conceptually relevant source material.

Following this premise, the search strategy was based on first defining seed terms that are likely to find relevant scales to inspire the development of our scale. These seed terms were as follows:

 + technology, analytics, “big data”, “artificial intelligence”, “data science” AND readiness OR maturity AND scale OR instrument

**Fig. 2:**
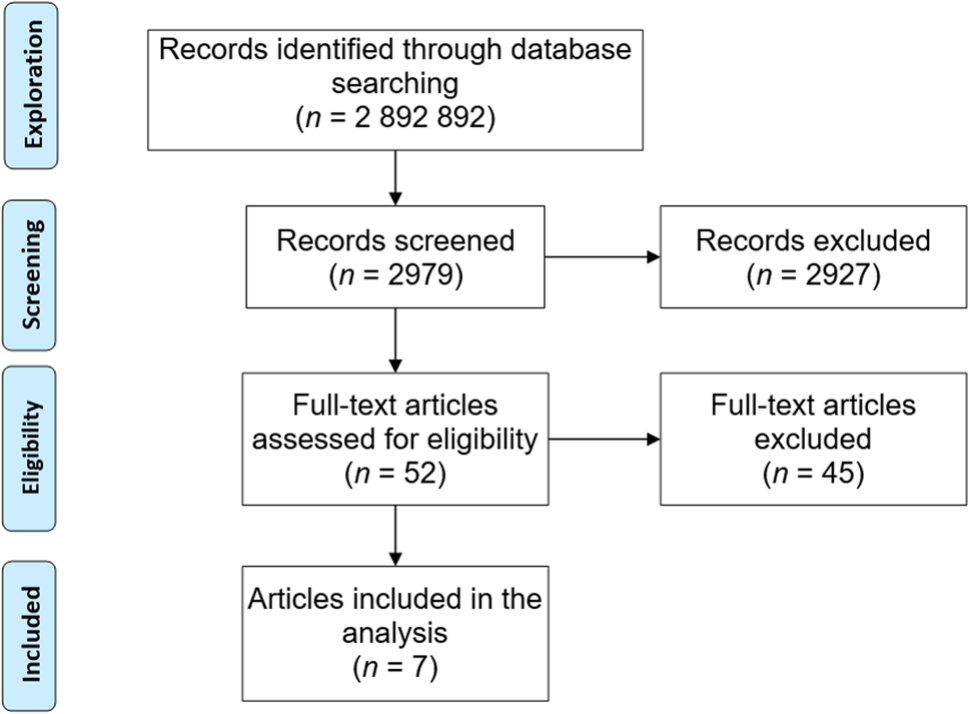
PRISMA-inspired (http://www.prisma-statement.org/) depiction of the literature review process

The seed terms were combined into different search phrases (e.g., + technology + readiness + scale), resulting in 20 such combinations (shown in Appendix 1^2^). Searches with these phrases were then conducted in Google Scholar and Science Direct. In total, Google Scholar yielded 2,734,310 results in total, while Science Direct yielded 158,582 results in total. We reviewed only the top results (i.e., those located in the first ~ ten search result pages; we found that increasing the number from this did not bring any more relevant results) for each search phrase because of the high number of articles located. The breakdown of the number of results per search and the number of screened results can be found in Appendix 1.^3^ In total, we screened 2,979 articles in a process that took several days of work from two researchers (Fig. 2 illustrates this process).

The screening was done by reviewing the abstract texts. Two researchers responsible for the screening looked for any mentions in the abstract that the article develops a technological readiness or maturity scale. Based on the screening, the overwhelming majority of the articles did not actually develop a scale but either applied one or presented conceptual ideas without mentioning empirical validation. In total, 52 articles were identified for full-text reading. Overall, the vast majority of the screened articles were found irrelevant, which implies that the literature searches could have been narrowed down more efficiently. However, in the end, we were able to find a satisfactory number of articles that provided the necessary inspiration for the development of the scale.

The corresponding full-text articles were then downloaded and reviewed. The inclusion or exclusion decisions were made case-by-case among two researchers that were responsible for this research step. We only included peer-reviewed full articles (e.g., no theses or workshop papers) that developed a scale for technology readiness or maturity focused on organizations (not on users or consumers) and included a full list of measurement items (not only examples) available in the article or in its appendices. The exclusion reasons are shown in Table 1. In total, 45 articles were excluded (86.5%), with seven articles (13.5%) remaining. Appendix 1^4^ shows the included and excluded articles.

**Table 1:** Reasons for excluding articles in eligibility assessment

Reason for exclusion	n	% of excluded
no items	32	71.1%
not peer-reviewed full paper	5	11.1%
exemplary items only	3	6.7%
not available for download	2	4.4%
focuses on consumers, not organizations	1	2.2%
does not correspond to our readiness definition	1	2.2%
duplicate from same authors	1	2.2%
Total	n = 45	86.5% of total assessed

### Development of constructs and items

We then recorded each construct (i.e., the phenomenon that the study measures) and item (i.e., a statement or question for organizational decision makers) from the qualified seven articles in a spreadsheet. The identified constructs (*n* = 42) and items (*n* = 155) were used as inspiration to create the PRS. This process included (a) removing redundant items that refer to the same idea and (b) modifying/rewriting the items so that their content is relevant for the concept of persona readiness. The inspirational constructs and items, along with their assessment of relevance for personas, can be seen in Appendix 1.^5^

## Scale dimensions and items

Table 2 shows the dimensions of the first version of the PRS [98]. The dimensions were adopted from previous scales [13, 61, 64, 86, 117], and fitted to the persona context. The final choice was based on internal discussion among the research team, where everyone could contribute to shaping the scale as a means of achieving face validity. Overall, the dimensions represent the different facets of persona readiness. The following subsections discuss each dimension.

**Table 2:** The dimensions of PRS (version 1, before the pilot study)

Readiness dimension	Description
Need readiness	This subscale measures the perceived need for personas and customer understanding in general
Culture readiness	This subscale measures the commitment to understand users and engaging in empathetic thinking
Knowledge readiness	This subscale measures the level of understanding concerning the concept of personas and their application in real use cases
Resource readiness	This subscale measures the financial, human, and support resources for the persona project
Data and systems readiness	This subscale measures the collection and richness of user data and associated systems
Capability readiness	This subscale measures the organization’s technical competence to create and maintain personas
Goal readiness	This subscale measures how well the organization sets goals for personas and monitors goal attainment with proper metrics

### Need readiness

The need readiness (NR) dimension and its items are inspired by the Strategic Readiness (SR) [64], Managerial Acquiescence (MA) [86], and Urgency to Change (UC) [61] constructs in related literature. An example item is, ‘Our organization needs personas’. Overall, NR implies that the organization has an awareness of the benefits of personas. This may not always be the case [42, 70, 78], as negative connotations (“bad reputation”) may be associated with the concept of personas among some stakeholders as a non-serious or non-useful tool [93], and management support may be lacking [77]. In contrast, organizations that are ready for personas perceive them as beneficial (at least potentially) and view personas as feasible to implement [61]. In other words, there is a recognized “need” for personas within the organization. Prior research postulates that the perceived need for technology can vary by organizational level [64]. This may also be the case for personas, as their potential benefits are associated with different job roles in the organizational hierarchy [92]. For example, senior management may perceive personas as important for strategic decisions; middle management for tactical decisions and planning; and operational staff (e.g., software developers, designers, and user support) for user-centric design choices in their daily work.

### Culture readiness

Culture readiness (CR) and its items are inspired by the Organizational Culture Readiness (OC) [61], Cultural Readiness (CL) [64], Culture (CU) [13], Customer Orientation (CO) [62], Market Orientation (MO) [117], and Developmental Culture (DC) [62] constructs in related literature. As can be seen from the large number of similar constructs, culture is widely recognized as an important antecedent to technology adoption. An example item is, ‘User understanding is crucial for us.’ As such, CR aims to capture the organization’s commitment to understanding users in *general*, i.e., their adherence to user-centered thinking [62]. Whereas NR focuses on personas, CR more broadly measures the degree of customer orientation. It is possible, for example, that the organization has a customer-driven culture, but they do not perceive the need for personas. Nonetheless, if they have a customer-centric culture, they are readier for personas than an organization that does not consider customer understanding important. CR contains the aspect of empathy that, as an integral part of the user-centric decision-making process, arises from the persona literature [26, 37, 69, 77]. The premise is that empathy is enhanced by personas and results in more user-centric (and therefore better) design and product development choices.

### Knowledge readiness

Knowledge readiness (KR) is inspired by the Cognitive readiness (CG) [64] and Employee Engagement (EE) [86] constructs in related literature that deal with possessing relevant information for effective decision-making. An example item is, ‘We know how to use personas.’ To this end, KR involves basic understanding of the concept of personas among the team members and experience in applying personas in real use cases. Lack of experience and know-how of personas can be detrimental to their application [95, 97] simply because any questions, doubts, and lack of reference examples hinder a stakeholder’s ability to make use of personas in a meaningful way. Furthermore, a lack of clarity on what personas are and how they are used can make them appear abstract, impersonal, and untrustworthy to stakeholders [70]. As such, a foundational understanding of the persona concept and the ways personas are used as design tools is required for a persona-ready organization.

### Resource readiness

Resource readiness (RR) and its items are inspired by the Resource Readiness (RR) [64], Employee Involvement (EI) [61], Partnership Readiness (PR) [64], Facilitating Conditions [110], and Training (TA) [13] constructs in related literature. An example item is, ‘Training is available for team members not familiar with personas.’ Overall, RR relates to the availability of crucial resources for the persona project along the persona lifecycle of the steps of creation, evaluation, and implementation [2]. Lifecycle thinking is important, as organizations might not properly follow through with persona application after their creation, instead of having an attitude of personas being a one-time analytical exercise [88, 89]. The consensus in the persona literature, which is also paralleled in technology adoption literature (e.g., CRM systems [45]), is that the mere set-up of a tool is not adequate to guarantee that relevant stakeholders will use it in their actual jobs. Hence, resources need to be directed to ensure successful creation and adoption. The necessary resources at different stages of the persona project may be provided by in-house personnel or an external consultancy. Moreover, the organization benefits from appointing one or more points of contact with the responsibility to ensure the success of the persona project, which includes ensuring that the personas are updated for the organization’s needs [52]. This person is sometimes characterized as a “persona champion” [71, 111]. Finally, formal training ought to be provided for the team members not familiar with personas, as major questions typically surround personas, involving aspects from their creation (e.g., “Where is this information coming from? Can I trust it?”) to their application (“How can I actually use this for my job?”).

### Data and systems readiness

Data and systems readiness (DR) and its items are inspired by the IT readiness (IT) [64], Technology compatibility (TC) [117], and Technological Orientation (TO) [62] constructs in related literature. An example item is, ‘We actively collect user data.’ Therefore, DR refers to activities supporting the creation of high-quality personas [23, 24]. Generating data-driven personas that are frequently updated is characterized by the repetitive collection of user data. When using large datasets of online users, organizations need to be able to wield big data for persona creation, characterized by volume, variability, veracity, and velocity [105]. In addition, the data has to satisfy the requirements of creating truthful and diverse persona sets that contain complete information to be helpful for decision-making tasks. Therefore, this “rounded persona” principle asserts stringent requirements on what variables to store and information to extract. The exact data requirements depend on the applied persona creation approach [48]. Typically, personas contain information about behaviors, demographics, goals, and needs [76, 91], therefore requiring that the organization has access to diverse and rich datasets about their users.

### Capability readiness

Capability readiness (BR) is inspired by the Big Data Capability (BC) [62], Data Analysis Expertise (DA) [13], Analytical Skills (AS) [85], and IT & Data Skills (DS) [85] constructs. An example item is, ‘We have advanced know-how on user segmentation.’ Overall, BR involves technical competence to operate systems and data required for data-driven persona generation [53]. This includes knowledge of algorithms, user data structures, databases, and external data sources such as APIs [53, 54], as well as a sound understanding of user segmentation principles and how these relate to statistical techniques such as dimensionality reduction or clustering [44] that are typically used for persona generation [5, 6]. As with data, the exact required capabilities depend on the applied persona creation approach (qualitative, quantitative, or mixed [74]).

### Goal readiness

Goal readiness (GR) and its items are inspired by the Measurement System Readiness (MS) [61], Policy Orientation (PO) [117], and Communication and Policy Application (CP) [13] constructs. An example item is, ‘We have clearly defined use cases for personas.’ Thus, GR refers to implementing tracking of performance outcomes. If personas are left unattended after their creation, the effort put into the project can easily be wasted [19, 24]. Personas also need to support achieving the team’s goals to make the team receptive to personas [88, 103]. For these reasons, performance metrics (e.g., marketing outcomes, user satisfaction) are required to gauge the success of the persona project. The metrics should be aligned with an implementation plan (i.e., a list of campaigns/projects/activities/programs where personas are to be applied, along with a description of who and by whom), and concrete goals (e.g., ‘deploying personas will improve user satisfaction by 15% within six months of the introduction of the finalized personas’).

### Measurement items before validation studies

Table 3 shows the twenty-two items of the PRS after the literature review and prior to pilot testing. The next research steps involve (a) a qualitative pilot study to clarify that the statements in the PRS make sense to participants (clarity, content) and (b) statistically testing that the items load appropriately to the proposed dimensions.

**Table 3:** PRS Version 1. Items marked with [D] were marked optional for qualitative personas, whereas items marked with [T] were marked optional for quantitative personas. Items with either were required in all cases. Mixed-method personas [84] may utilize all statements

Item	NR	CR	KR	RR	DR	BR	GR
Our organization needs personas	x						
We consider personas important	x						
Personas would be useful for us	x						
We need personas now	x						
User understanding is crucial for us		x					
Empathy is required for understanding users		x					
Most of the people in our organization know what a persona is			x				
Most of the people in our organization have used personas in their work			x				
We know how to use personas			x				
We have a person in our organization who is strongly advocating for personas				x			
We have a dedicated budget for persona creation and implementation				x			
Training is available for team members not familiar with personas				x			
We actively collect user data. [D]					x		
We have extensive user data, including behavioral and demographic information					x		
Our user data is frequently updated. [D]					x		
Our user data is rich, including user interviews or written feedback. [T]					x		
We have data science expertise. [D]						x	
We have advanced know-how on user segmentation						x	
We have a plan for implementing personas after their creation							x
We have quantitative goals for persona use							x
We have clearly defined use cases for personas							x
We have defined quantitative metrics to measure the results of persona use							x

## Validation studies

When developing a new scale, researchers may have preconceptions of how items will be structured beforehand; nonetheless, various types of testing are needed to determine the number of latent factors and the structure of the items [79]. Therefore, after devising the constructs and items for the initial version of the PRS, we conducted several validation steps (see Table 4). The steps rely on mixed methods, i.e., both qualitative and quantitative techniques are used [109] to increase the robustness of the results.

**Table 4:** Validation approaches of the study

Validity type	Question to address	Approach taken			
		Internal discussions	Literature review	Qualitative pilot study	Statistical testing/calculations
Face	Does the PRS appear to be suitable to its aims?	x		x	
Content	Is the PRS fully representative of the phenomenon it aims to measure?		x	x	
Construct	Does the PRS measure the phenomenon that it intends to measure?				x
Nomological	Does a higher persona readiness correlate with the successfulness of a persona project?				x

Validation proceeds in several stages. First, we carry out a *qualitative pilot study* (PILOT SAMPLE, *n* = 12) in order to evaluate if the items are suitable and/or if some aspects of persona readiness are missing. Second, we carry out a quantitative evaluation study to examine the scale validity from a statistical point of view. This involves conducting (a) an *exploratory factor analysis* (EFA) to determine a fitting solution of factors and items (EXPLORATORY SAMPLE, *n* = 125) and (b) a *confirmatory factor analysis* (CFA) to assess the applicability of the scale on yet another sample (CONFIRMATORY SAMPLE, *n* = 247). The use of multiple steps and independent samples increases the robustness of the validation. Finally, the test the nomological validity – whether the scale correlates logically with constructs with which it is supposed/likely to be correlated (Yi and Gong, 2013) – by calculating the correlation of the scale and subscales with the scale measuring the perceived successfulness of a persona project.

## Pilot sample: qualitative pilot study

### Participants

A pilot study was conducted to confirm whether the questions in the PRS make sense, i.e., to achieve construct validity (that the PRS indeed measures persona readiness) and content validity (that the PRS is not missing critical aspects). In this process, we investigated the overall structure of the survey and obtained modifications based on the feedback from participants experienced with personas and considered as experts in this context.

The participants for the pilot study were recruited by leveraging professional networks, cold messaging on a professional social network (LinkedIn), and using the initial interviews to snowball additional participants. Applying these techniques yielded a total of 12 participants (R), ranging from UX designers to executives (see Table 5). These individuals were considered eligible based on the following criteria: (a) the participant is currently working with or has worked with personas, (b) the total roster of participants has diversity in terms of gender (7 were females, 58.3%), age (M = 37.2, SD = 8.7), domain, and (c) the level at which the participants work with personas is varied, including operational (*n* = 5), tactical (*n* = 5), and strategic (*n* = 2) decision making.

**Table 5:** Participant information (SAMPLE 1)

	Age	Gender	Job position	Industry^a^
R1	45–55	Male	Design Community Lead	Logistics and Supply Chain*
R2	45–55	Male	Head of Business Processes, Director	Information Technology and Services
R3	25–35	Female	Product Design Manager	Information Technology and Services
R4	25–35	Male	Head of Design	Financial Services
R5	45–55	Female	Digital Communications Manager	Dairy*
R6	25–35	Female	Industrial Researcher	Computer Software
R7	35–45	Female	Customer Insights Manager	Logistics and Supply Chain*
R8	45–55	Male	Lead Product Design Strategist	Logistics and Supply Chain*
R9	25–35	Male	UX Designer	Dairy*
R10	25–35	Female	UX Designer	Dairy*
R11	25–35	Female	UX Designer	Dairy*
R12	25–35	Female	Consultant	Machinery

^*^Indicates the same organization

^a^Categorization provided by LinkedIn

### Procedure

The participants were invited for 30-min individual interviews (M = 30.2, SD = 4.5 min) over Microsoft Teams. The interview format was based on the think-aloud method [31] combined with a semi-structured interview [39]. The participants shared their screens, and as they went through the survey, they would tell us what the questions made them think of and why they answered the way they did. After the questionnaire, we posed follow-up questions about the survey to get a sense of what was good, and what could be improved, as well as asking the participants for their own opinion on their organization’s persona readiness. Content validity (i.e., the extent to which the scale represents all facets of a given construct) was assessed by asking the participants if the scale was missing something important for measuring persona readiness in organizations. The interviews were recorded and transcribed verbatim, and the transcriptions were used as the foundation of an affinity diagram where we grouped the statements from the interviews based on their similarities [106]. This served as the basis for the improvement of the survey. Furthermore, we tabulated the results of each participant to see how they performed on the PRS (see Appendix 2^6^).

### Results

Based on the feedback from the pilot study participants, several modifications were made to the survey. These modifications were based on the insights which were given both during the survey, as well as the follow-up interview. In practice, the researchers that were in charge of the pilot study reported their findings to the other researchers, and the proposed modifications were discussed one at a time. Table 6 shows the PRS after the pilot study modifications. Thereafter, we explain the main reasoning behind the changes.

**Table 6:** PRS Version 2 (after pilot study)

ID	Item
CR1	Customer understanding is a strategic priority for us
CR2	We want to develop empathetic understanding of our customers
CR3	Our organization needs personas
CR4	Several people in our organization consider personas important
CR5	Personas help reach our organization's goals
CR6	Executives in my organization have made personas a priority
KR1	Most of our people that work in positions where customer understanding is relevant to their work know what a persona is
KR2	Most of our people for whom customer understanding is relevant have used personas in their work
KR3	We know how to use personas
RR1	We have one or more people in our organization who are strongly advocating for personas
RR2	We have a sufficient budget for persona creation and implementation
RR3	Formal training on how to use personas is available for team members that are not familiar with personas
DR1	We actively collect customer data that could be used for persona creation
DR2	We have behavioral and demographic data about our customers
DR3	Our customer data is frequently updated (frequently means at least monthly)
DR4	We collect information about our customers both quantitatively (e.g., web analytics) and qualitatively (e.g., interviews)
BR1	There is expertise in our organization that is helpful for persona creation
BR2	We have a high level of skill on customer segmentation
BR3	We have one or more people who are responsible for implementing personas
BR4	We know how to create personas
GR1	We have a plan for how to use personas after their creation
GR2	We have defined specific goals for persona use
GR3	We have created specific use cases for personas
GR4	We use quantitative metrics to measure projects that personas are part of
GR5	We use quantitative metrics to directly measure the results of persona use

Even though it was stated that the participants were supposed to answer based on their entire organization, many were confused by this (e.g., R1, R3, R5, R6, R7, R9, R11). This was particularly highlighted in the cases where the primary working force in the organization was workers on the ground floor, like dairy workers, farmers, or ship crews (e.g., R1, R5, R7, R8, R8, R10, R11). In these cases, the participants would sometimes place their answer somewhere in the middle, for example, by concluding that if someone in my organization never uses personas (Strongly Disagree), but my department uses personas a lot (Strongly Agree), then my answer must be somewhere in the middle (Neutral). To address this issue, a definition for the organization was added: “When answering the questions, choose the organization level you are most familiar with. This could be a team, department, division, or the whole company.”

Pilot study participants were all familiar with personas, but this might not be the general case for everyone completing the survey. Therefore, a persona definition was provided, with an example of what a persona could be like: “*Personas are fictional persons representing a group of similar users or customers of a product or service. For example, ‘Loyal Larry’ could represent a loyal middle-aged customer who habitually buys your product when visiting a supermarket*.” Despite the fact that the PRS is primarily targeted at organizations that do not yet use personas, to scope their readiness, it is possible that organizations that already have created personas or used them in the past will take the survey. This information can be valuable for later analysis, so we added a question about the current status of the organization, where participants can select one or several options: “Our organization is planning to create personas.”; “Our organization has already created personas.”; “Our organization is actively using personas.”; “None of the above.” In addition, we ask if the respondent thinks their answers apply to themselves only, their team only, their department or division only, or the whole organization.

None of the participants we had recruited worked with personas in a quantitative manner; therefore, there was confusion surrounding how they would even consider quantitative goals. Therefore, we revised GR02 by removing the word “quantitative”, as persona use can have any type (also qualitative) of goal. We split GR04 into two separate statements, one addressing the measurement of efforts that personas are a part of and the other asking if the organization directly measures the effect of persona use (GR4 and QR5 in Table 6).

The items had initially been provided in one list in randomized order. However, several participants found the order of items to be confusing, which led us to disable randomization in the final implementation. In addition, we divided the items into separate sections and provided reasoning for them (i.e., the definition of the dimension), to address the lack of context that some participants expressed. As a result, completing the survey was considered more fluent and logical. To address this issue, we added a new question that is not part of the PRS but is still asked from the respondents as background information, namely, “Please choose the option that best describes your organization.”. Through this, we can examine how different maturity levels affect PRS scores. For example, it is possible that an organization has already created personas but is not really ready for them. Our survey would ideally show this discrepancy, helping to explain the results of the persona project.

## Validation SAMPLES: quantitative validation studies

We collected two independent, non-overlapping samples for the quantitative scale validation, referred to as the EXPLORATORY SAMPLE and the CONFIRMATORY SAMPLE. The EXPLORATORY SAMPLE was used for exploratory factor analysis, and the CONFIRMATORY SAMPLE was used for confirmatory factor analysis. The two samples were collected to enable exploratory and confirmatory factor analyses on separate samples. In the following section, we describe these samples.

### Participants

#### Recruitment

The purpose of the validation study was to statistically evaluate the scale’s reliability and validity. For both samples, a carefully selected number of participants from the online survey platform Prolific was recruited. Prolific has been used in several persona user studies in the past [94, 96, 99, 100], and its data quality has been found satisfactory for academic research [82, 83]. We applied custom prescreening to increase the validity of the responses with the following sampling criteria:

- Minimum Age: 23, Maximum Age: 62 (inclusive)—the purpose was to focus on those in active work life
- United Kingdom, United States, Ireland, Australia, and New Zealand—the purpose was to focus on predominantly English-speaking countries to avoid misunderstanding of the questions
- Student Status: No—the purpose was to focus on those in active work life
- Excluding self-employed individuals—the purpose was to focus on people working in organizations larger than one person
- Employment status: Full-time—the purpose was to focus on people actively engaging in work life on a full-time basis
- Organizational tenure: excluding those with less than five months—the purpose was to focus on people that have an adequate understanding of their organization; hence a minimum tenure was required.

Piloting the PRS among the research team showed an average response time of approximately 15 min. Based on this estimate, we set the compensation rate in Prolific. To offer the participants a fair compensation for their time, we set a reward that exceeded the minimum National Living Wage for those aged 23 and over in the United Kingdom (based on the rate of April 2021^7^). To set the sample size for the CONFIRMATORY SAMPLE, we applied the common rule of thumb of 10:1 person-to-item ratio [114]. As there were 25 items, 250 respondents were recruited from Prolific. We collected a smaller number of 125 participants for the EXPLORATORY SAMPLE, as confirmatory factor analysis tends to require more participants to show meaningful results [57] relative to exploratory. We ensured that no participants were included in the two samples by first collecting the EXPLORATORY SAMPLE, and then excluding the participants in this sample (based on their Prolific ID) from the data collection job of the CONFIRMATORY SAMPLE. Three participants in the CONFIRMATORY SAMPLE failed an attention check question, and were removed, leaving *n* = 247 for the analysis. No participants from the EXPLORATORY SAMPLE were removed.

#### Description

In the following, the inline figures report the EXPLORATORY SAMPLE, and the figures in parentheses are for the CONFIRMATORY SAMPLE. Seventy-four (59.2%) participants were female (CONFIRMATORY SAMPLE: *n* = 125, 50.6%). The average age of the participants was 39.8 years (SD = 9.4) (CONFIRMATORY SAMPLE: M = 39.5, SD = 9.2). In other words, the demographic composition of the samples was similar. The participants’ average work experience in their current company was 12.1 years (SD = 7.8) (CONFIRMATORY SAMPLE: M = 13.6, SD = 8.7). In the CONFIRMATORY SAMPLE, around half (47.0%) of the participants had used personas or were still using them. In contrast, the EXPLORATORY SAMPLE had relatively fewer people experienced with personas (see Table 7).

**Table 7:** Participants’ experience with personas

	EXPLORATORY SAMPLE		CONFIRMATORY SAMPLE	
	n	Proportion	n	Proportion
Experienced—have used personas before or am still actively using them	48	38.4%	**116**	**47.0%**
Beginner—did know about personas prior to taking this study, but hadn’t used them	**51**	**40.8%**	93	37.7%
Novice—did not know about personas prior to taking this study	23	18.4%	31	12.6%
Expert—have used personas and been part of creating them	3	2.4%	7	2.8%
N	125	100%	247	100.0%

The largest classes are bolded

**Fig. 3:**
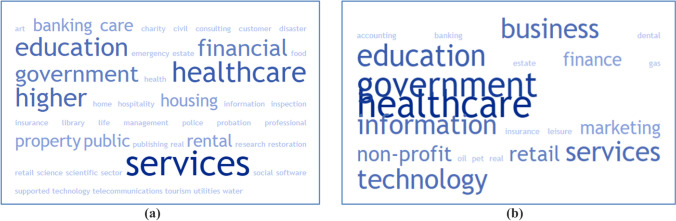
Word clouds representing the industries where the participants belonging to the EXPLORATORY SAMPLE **(a)** and the CONFIRMATORY SAMPLE **(b)** work

The industries where the participants were working were varied (see Fig. 3), but focused on education, healthcare, retail, government, and information technology (for both samples). The EXPLORATORY SAMPLE has a slightly more pronounced representation of software and banking, whereas the CONFIRMATORY SAMPLE has more participation from social care and local government. However, as a whole, both samples contain professionals from many fields.

In both samples, most participants were working for large enterprises (see Table 8). Job titles were highly varied, representing dozens of different positions. In the EXPLORATORY SAMPLE, the most common job titles included Manager (*n* = 37, 29.6%), Assistant (*n* = 10, 8.0%), Administrator (*n* = 7, 5.6%), and Officer (*n* = 6, 4.8%). In the CONFIRMATORY SAMPLE, the most common job titles included Manager (*n* = 61, 24.7%), Head of Department (*n* = 10, 4.0%), Assistant (*n* = 10, 4.0%), Analyst (*n* = 9, 3.6%), Supervisor (*n* = 8, 3.2%), and Engineer (*n* = 7, 2.8%), with the rest of the participants working in various other occupations (see Table 9). In both samples, close to a third of the participants indicated that their organization had created and was using personas (see Table 10). Roughly the same number of participants had not created nor planned to create personas. Around one-fifth of the participants were either planning to create personas or had created personas but were not actively using them.

**Table 8:** Participants’ organization sizes based on Eurostat classification (https://ec.europa.eu/eurostat/statistics-explained/index.php/Glossary:Enterprise_size)

	EXPLORATORY SAMPLE		CONFIRMATORY SAMPLE	
	n	Proportion (%)	n	Proportion (%)
Large enterprise: 250 employees or more	**73**	**58.4**	**162**	**65.6**
Medium-sized enterprise: 50 to 249 employees	29	23.2	53	21.5
Small enterprise: 10 to 49 employees	18	14.4	27	10.9
Microentreprise: 1 to 9 employees	5	4.0	5	2.0
N	125	100	247	100

The largest classes are bolded

**Table 9:** Most common job titles

	EXPLORATORY SAMPLE		CONFIRMATORY SAMPLE	
Job title	n	Proportion (%)	n	Proportion (%)
Manager	**37**	**29.6**	**61**	**24.7**
Assistant	**10**	**8.0**	10	4.0
Administrator	**7**	**5.6**	**15**	**6.1**
Officer	6	4.8	**17**	**6.9**
Head of Department	3	2.4	10	4.0
Consultant	3	2.4	2	0.8
Analyst	2	1.6	9	3.6
Supervisor	2	1.6	8	3.2
Engineer	2	1.6	7	2.8
Other	53	42.4	111	44.9

The three most frequent are bolded for both samples

**Table 10:** Current status with personas in the participants’ organizations

	EXPLORATORY SAMPLE		CONFIRMATORY SAMPLE	
	n	Proportion (%)	n	Proportion (%)
Our organization has created and is actively using personas	37	29.6	72	29.1
Our organization is planning to create personas	24	19.2	53	21.5
Our organization has already created personas	26	20.8	49	19.8
None of the above	**38**	**30.4**	**73**	**29.6**
N	125	100	247	100

The highest values highlighted

The participants were asked to name the organization they work for so that we could ascertain how many different organizations the sample has. Out of the combined number of participants in both samples (*n* = 372), 348 (93.5%) named their organization. We sorted the organization names alphabetically to identify duplicates. As a result, 15 organizations had two participants, one organization had three participants, and one organization had 10 participants; the rest of the organizations had one participant. Therefore, the number of unique organizations was: 348 − ((30 − 15) + (3 − 1) + (10 − 1)) = 348 − 26 = 322. However, given that there were 24 participants (6.5% of the total) that refused to disclose their organization, we can presume that some of these might have been the same organizations mentioned by the other participants. The proportion of duplicates among the non-disclosed participants can be estimated using the fraction of deduplicated organizations over the total number of organizations: (348 − 322)/348 = 7.47%. Applying this factor to the total number of participants in the combined sample, along with dropping the duplicate organizations, yields: 372 − 26 −  (0.0747 * 24) = 344.2 ≈ 344 organizations, which we estimate as the number of participating organizations.

### Procedure

The participants in both the EXPLORATORY SAMPLE and the CONFIRMATORY SAMPLE were shown the refined PRS statements (Table 6). The participants responded by expressing their agreement with each statement using a five-point Likert scale that ranged from 1 (Strongly disagree) to 5 (Strongly agree) with the additional option “Do not know”. The “Do not know” option should not have any impact on the calculated scores. In other words, when composite scores are computed, selecting “Do not know” will not interfere with the calculation so long as means are used rather than sums [28]. For the purposes of the validation exercise, “Do not know” (26.61% of the participants had selected it in one or more items in the EXPLORATORY SAMPLE, and 21.05% in the CONFIRMATORY SAMPLE) were imputed using Markov Chain Monte Carlo (MCMC) imputation [115] to preserve as much data as possible.

### EXPLORATORY SAMPLE: exploratory factor analysis

The exploratory factor analysis (EFA) was conducted on the entire pool of questions, using the designated EFA sample and Principal Component estimation. As it was expected that some degree of correlation would emerge between the factors, an oblique rotation method—Direct Oblimin—was used [1, 41, 67]. We began by evaluating the data adequacy for the purposes of EFA. First, the normality of the data was assessed for each item through their skewness and kurtosis. All of them were under the absolute value of 3, indicating that all items had a sufficiently normal distribution [57], as shown in Table 11.

**Table 11:** Descriptive statistics for individual items

Item	Min	Max	Mean	SD	Skewness	Kurtosis
CR1	2	5	4.59	0.568	− 1.299	2.223
CR2	2	5	4.54	0.628	− 1.460	2.806
CR3	1	5	3.58	0.996	− 0.476	− 0.093
CR4	1	5	3.77	1.128	− 0.859	− 0.012
CR5	1	5	3.70	1.102	− 0.738	− 0.079
CR6	1	5	3.13	1.192	− 0.151	− 0.823
KR1	1	5	3.58	1.155	− 0.663	− 0.433
KR2	1	5	3.53	1.199	− 0.632	− 0.567
KR3	1	5	3.38	1.117	− 0.605	− 0.42
RR1	1	5	3.31	1.312	− 0.357	− 1.015
RR2	1	5	3.22	1.131	− 0.360	− 0.638
RR3	1	5	2.52	1.082	0.262	− 0.913
DR1	1	5	3.57	1.215	− 0.680	− 0.498
DR2	1	5	3.82	1.219	− 0.860	− 0.318
DR3	1	5	3.63	1.207	− 0.594	− 0.685
DR4	1	5	3.78	1.109	− 0.963	0.217
BR1	1	5	3.54	1.083	− 1.006	0.432
BR2	1	5	3.73	1.070	− 0.888	0.278
BR3	1	5	3.21	1.296	− 0.348	− 0.982
BR4	1	5	3.37	1.152	− 0.570	− 0.364
GR1	1	5	3.22	1.233	− 0.431	− 0.916
GR2	1	5	3.13	1.245	− 0.347	− 0.956
GR3	1	5	3.24	1.301	− 0.402	− 1.054
GR4	1	5	3.19	1.225	− 0.317	− 1.046
GR5	1	5	3.03	1.193	− 0.190	− 1.036

Second, we evaluated Kaiser–Meyer–Olkin (KMO), which yielded a value of 0.919, and additionally, Bartlett’s test of sphericity was significant (χ^2^(300) = 2537.411, *p* < 0.001), indicating that the data is adequate for EFA [41, 67]. A final evaluation was done for each item through their Measures of Sampling Adequacy (MSA), obtained through the main diagonal of the anti-image matrix—all of them were above the 0.50 threshold, and as such, none were candidates for removal [41]. In order to ascertain the optimal number of factors, three criteria were employed: (a) Kaiser’s criterion (> 1 eigenvalues), (b) extracted variance, and (c) scree plot interpretation. The first two can be seen in Table 12. Accordingly, a single factor accounted for 50.9% of variance, meeting the minimum threshold of 50% [67], and as such, this criterion contributed little to determining the optimal number of factors. Visual inspection of the scree plot revealed a sharp inflection point at the two-component mark, and another one to a lesser degree at the four-component mark, indicating these as potential solutions. Finally, Kaiser’s criterion points towards a four-factor solution. Therefore, we explored this solution first, which is shown in Table 13.

**Table 12:** Summary of eigenvalues and extracted variance

Component	Eigenvalues	% of Variance	Cumulative %
1	12.721	50.883	50.883
2	1.888	7.552	58.435
3	1.299	5.194	63.630
4	1.190	4.761	68.391
5	0.968	3.870	72.261
6	0.843	3.373	75.634

Table is truncated at 6 components since further solutions beyond this threshold were not considered. Original table considered up to 25 components (explaining 100% of variance)

**Table 13:** Exploratory Factor Analysis (4 factors)

Item	Factor			
	1	2	3	4
GR2	0.901			
GR1	0.883			
GR4	0.880			
GR5	0.860			
BR3	0.832			
GR3	0.811			
BR4	0.797			
KR3	0.777			
KR1	0.777			
KR2	0.763			
RR1	0.756			
RR3	0.655			
BR1	0.613			
CR4	0.603			− 0.327
CR6	0.517			− 0.476
BR2	0.492		0.358	
RR2	0.484			
DR4		0.846		
DR2		0.743		
DR3		0.742		
DR1		0.691		
CR2			0.807	
CR1			0.762	
CR3				− 0.705
CR5	0.433			− 0.592

An EFA with Direct Oblique rotation is shown (pattern matrix). Loadings under 0.30 are omitted

**Table 14:** Exploratory Factor Analysis (6 factors)

Item	Factor					
	1	2	3	4	5	6
GR4	0.917					
GR5	0.907					
GR3	0.793					
GR1	0.787					
GR2	0.735					
BR3	0.593					
BR2	0.517		0.362			
BR4	0.425				0.416	
DR3		0.827				
DR4		0.807				
DR2		0.677				
DR1		0.596		− 0.382		
CR2			0.801			
CR1			0.751			
CR3				− 0.835		
CR5				− 0.728		
CR6				− 0.609		
CR4	0.314			− 0.454		
KR1					0.911	
KR3					0.793	
KR2					0.675	
BR1					0.359	
RR1					0.330	
RR2						0.905
RR3						0.553

An EFA with Direct Oblique rotation is shown (pattern matrix). Loadings under 0.30 are omitted

The 4-factor solution, which explained 68.4% of variance, departed substantially from the theoretically expected and designed structure, with items from various dimensions coalescing into a singular factor (with the notable exception of DR—Data and systems Readiness. Some items with low loadings and relevant cross-loadings were also noted. This solution was deemed impracticable due to its substantial disconnection from the underlying theory; as such, and as the scale was designed to accommodate six dimensions, a forced extraction of 6 factors was attempted. This solution, explaining 75.6% of the variance is shown in Table 14.

**Table 15:** Fit evaluation for each model

Model	χ^2^/df	CFI	PCFI	RMSEA	AIC	BCC
I	3.738	0.866	0.750	0.105	1101.840	1117.204
II	4.023	0.893	0.752	0.111	743.744	753.078
III	2.450	0.950	0.780	0.077	490.244	500.324
IV	1.829	0.974	0.777	0.058	321.109	329.312
V	1.820	0.974	0.789	0.058	319.656	327.524

The six-factor solution was equally problematic; Capability Readiness (BR) loaded into the same factor as Goal Readiness (GR), albeit with unsatisfactory loadings, whereas Culture Readiness (CR) was spread over various factors with cross-loadings. However, steps could arguably be taken to produce a statistically valid structure for Confirmatory Factor Analysis, based on either the four- or the six-factor solution; both of those departed significantly from the theory-supported constructs. As good practices dictate that factor analysis should not be disconnected from empirical considerations [57, 68], and it is not uncommon for EFA structures to be dropped at the confirmatory stage [68], it was opted not to pursue an EFA-derived solution,^8^ but rather attempt a CFA using the designed structure, leaving open the possibility of falling back to an exploratory approach to the CFA if required in order to attain a final solution which is both statistically sound and theory-compatible.

### CONFIRMATORY SAMPLE: confirmatory factor analysis

#### Procedure

Confirmatory factor analysis (CFA) is a statistical technique used to verify the factor structure of a set of observed variables [57]. Model estimation was done using Maximum Likelihood, the most common option and robust to potential deviations from normality [11, 68]. CFA models are generally evaluated based on various fit indices [43]. For this analysis, we employed the χ^2^ goodness-of-fit test [14] and its χ^2^ statistic [16], the χ^2^/df index [11], the comparative-fit index (CFI) [15], the parsimony-adjusted variant of CFI—PCFI [68]—the root-mean-square error of approximation (RMSEA) [104], and finally, for adjudging improvements across model iterations, we employ Akaike’s Information Criterion (AIC) [7] and the Browne-Cudeck Criterion (BCC) [68]. As mentioned in the previous section, it was opted to not conduct a specification of the EFA-extracted structure and instead to employ the designed structure and a more exploratory-oriented strategy. In the following sections, the steps taken from the first to the final model will be described in detail for each iteration. Only the first model can genuinely be considered a confirmatory analysis, since it is the unchanged baseline model.

#### Model I

The baseline model consists of the full instrument, using the designed structure. With no changes, the fit was deemed as acceptable—with the exception of RMSEA—but with room for improvements (χ^2^(260) = 971.840, *p* < 0.001; χ^2^/*df* = 3.738; CFI = 0.866; PCFI = 0.750; RMSEA = 0.105; P[rmsea ≤ 0.05] < 0.001).

#### Model II

Moving into the following iteration, two changes were made. First, items with loadings under 0.50 in their respective factors were removed, as they threaten factorial validity [68]. Only two items were under this threshold—CR1 (“Customer understanding is a strategic priority for us.”), and CR2 (“We want to develop empathetic understanding of our customers.”). A subtler yet critical threat to model validity was detected in this baseline iteration. The RR scale exhibited a standardized correlation with BR of 1.02, above the theoretical plausible maximum value of 1. This indicates a Heywood case scenario [58] that required immediate addressing before any subsequent iteration. Heywood cases are typically caused by small sample sizes (which is not the case, as we met the recommended minimums) or model misspecification [57].

In this case, the most plausible explanation was excessive multicollinearity with the remaining dimensions. Although constraints could be placed to remediate the issue from a purely statistical point of view, this would not address the underlying cause, and the issue would likely re-emerge again in later models under the guise of convergent or divergent validity issues. As such, it was opted to remove the RR scale entirely as we proceeded into the next iteration. Model fit remained qualitatively unchanged, despite some minor shifts in some of the indicators (χ^2^(160) = 643.744, *p* < 0.001; χ^2^/*df* = 4.023; CFI = 0.893; PCFI = 0.752; RMSEA = 0.111; P[rmsea ≤ 0.05] < 0.001).

#### Model III

In this iteration, we conducted model optimizations aimed at fit improvement. To this end, Modification Indices (MI) [11, 20] were analyzed in order to identify opportunities for ameliorating the model’s fit. A threshold of 11 or higher was defined for the MIs, corresponding to a Type I error probability of 0.001 [68]. Only plausible MI changes were considered—notably, specification of covariances between error terms for manifest variables loading into the same factors, whenever these yielded a positive fit gain. Three such covariances were specified in the GR scale. As a result, substantial improvements were seen regarding model fit (χ^2^(156) = 382.244, *p* < 0.001; χ^2^/*df* = 2.450; CFI = 0.950; PCFI = 0.780; RMSEA = 0.077; P[rmsea ≤ 0.05] < 0.001). Although this degree of fit could be considered sufficient, we opted to continue with further refinements of the model.

#### Model IV

In this iteration, we continued the exploration of MI opportunities for improvement. No valid covariances remained at the 11 thresholds, so we now checked for non-valid changes—i.e., covariances between error terms of manifest variables belonging to different factors. These are typically indicative of cross-loadings for a given item, and although specifying an inter-factor covariance is not a valid change, deleting an item with substantial cross-loadings can be considered [57]. BR3 (“We have one or more people who are responsible for implementing personas.”) and BR4 (“We know how to create personas.”) exhibited such cross-loading behavior relative to the KR scale. As such, both items were removed. This resulted in immediate gains to the model fit, putting it comfortably within the qualitative threshold of “good” (χ^2^(122) = 224.929, *p* < 0.001; χ^2^/*df* = 1.844; CFI = 0.973; PCFI = 0.776; RMSEA = 0.059; P[rmsea ≤ 0.05] < 0.001). As no further improvements could be easily made to the model fit, we proceed into the validity checks phase. A minor issue emerged regarding the discriminant validity of the CR scale due to a high degree of correlation between it and the GR latent variable (r = 0.875). Notably, the square root of the average variance extracted for CR was less than the absolute value of that correlation, and simultaneously its average variance extracted was less than the maximum shared variance.^9^ Although this issue could likely be ignored, we opted to address it for robustness’ sake, and as such, we proceeded into the final iteration.

#### Model V

**Fig. 4:**
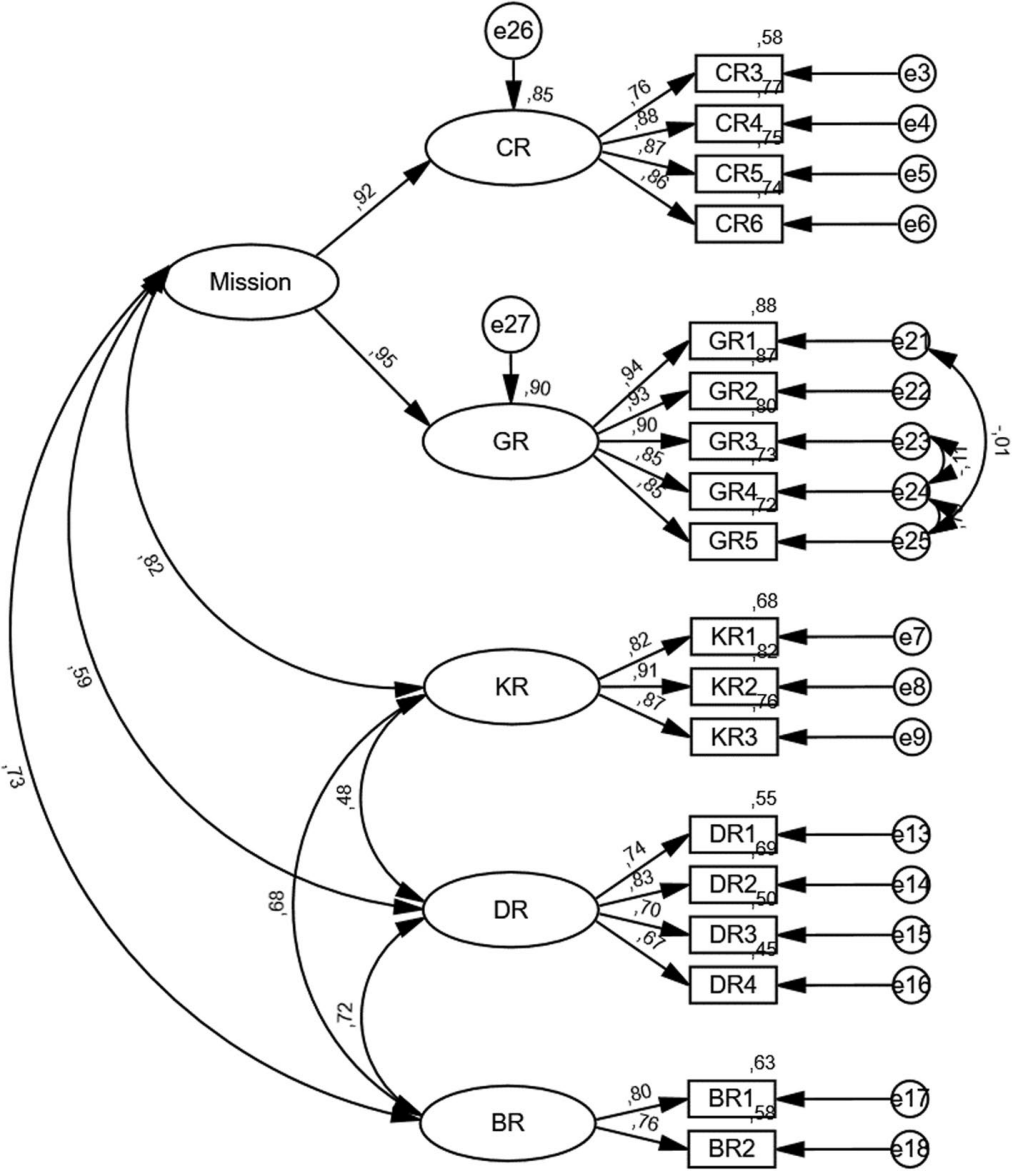
CFA Model for the final iteration of the scale (Model V)

In order to address the discriminant validity issue, and due to the existence of a substantial correlation between the CR and GR scales, we explored whether a second-order latent variable could conceivably be introduced into the model, encompassing these two variables. As such, in this iteration, we created a second-order latent variable—which we named “Mission” (MN), due to the semantic content of the items in each sub-scale. This second-order latent variable loaded robustly into both CR (r = 0.92) and GR (r = 0.95). Furthermore, it yielded slight increases to the model fit (χ^2^(124) = 225.656, *p* < 0.001; χ^2^/*df* = 1.820; CFI = 0.974; PCFI = 0.789; RMSEA = 0.058; P[rmsea ≤ 0.05] < 0.001). Finally, it remedied the validity issue, which will be noted in the following section. As such, we settled on Model V as the definitive model. Table 15 summarizes the fit changes for each step of this exercise; Table 16 includes the factorial loadings for the final instrument, and Fig. 4 illustrates the final model.

**Table 16:** Factorial loadings for the final model

Code	Item content	Loading
CR3	Our organization needs personas	0.760
CR4	Several people in our organization consider personas important	0.876
CR5	Personas help reach our organization’s goals	0.866
CR6	Executives in my organization have made personas a priority	0.863
KR1	Most of our people that work in positions where customer understanding is relevant to their work know what a persona is	0.823
KR2	Most of our people for whom customer understanding is relevant have used personas in their work	0.905
KR3	We know how to use personas	0.872
DR1	We actively collect customer data that could be used for persona creation	0.740
DR2	We have behavioral and demographic data about our customers	0.829
DR3	Our customer data is frequently updated (frequently means at least monthly)	0.704
DR4	We collect information about our customers both quantitatively (e.g., web analytics) and qualitatively (e.g., interviews)	0.667
BR1	There is expertise in our organization that is helpful for persona creation	0.795
BR2	We have a high level of skill on customer segmentation	0.761
GR1	We have a plan for how to use personas after their creation	0.937
GR2	We have defined specific goals for persona use	0.932
GR3	We have created specific use cases for personas	0.897
GR4	We use quantitative metrics to measure projects that personas are part of	0.853
GR5	We use quantitative metrics to directly measure the results of persona use	0.847

**Table 17:** Validity and reliability evaluation

	CR	AVE	MSV	ASV
KR	0.901	0.752	0.677	0.457
DR	0.826	0.544	0.524	0.369
BR	0.754	0.606	0.531	0.507
MN	0.934	0.876	0.677	0.521

KR	DR	BR	Mission
Correlations			
0.867			
0.477	0.737		
0.682	0.724	0.778	
0.823	0.595	0.729	0.936

The diagonal of the correlation matrix indicates the square root of the AVE

We evaluated three facets of validity for the final model—factorial, convergent, and discriminant, as generally suggested for scale validation [41, 68]. *Factorial validity* is attained when all standardized loadings are above the 0.50 threshold [68], which was already assured during the second iteration of the model. The second aspect, *convergent validity*, generally requires high loadings for each specific construct [32]. This is evaluated through the Average Variance Extracted (AVE), which is given by Eq. 1:

$$\widehat{{AVE}_{j}}= \frac{\sum_{i=1}^{k}{\lambda }_{ij}^{2}}{\sum_{i=1}^{k}{\lambda }_{ij}^{2}+ \sum_{i=1}^{k}{\varepsilon }_{ij}}$$

The AVE must exceed the threshold of 0.50 for all factors to confirm convergent validity [68]—this was also confirmed for all factors after the final iteration of the model. The next facet is *discriminant validity*, which requires a low degree of inter-factor correlations and cross-loadings. This is demonstrated when the square root of the AVE for a given pair of factors is equal to or greater than the correlations between those factors; additionally, the AVE must be equal to or greater than both the Maximum Shared Variance (MSV) and the Average Shared Variance (ASV) [32, 41, 68]. As before, all factors met this criterion, thus confirming the scale’s discriminant validity. We proceeded by evaluating the scale’s reliability, which concerns its consistency. For this purpose, we employed the composite reliability (CR) indicator [32], which for a factor *j* with *k* items is given by Eq. 2:

$$\widehat{{CR}_{j}}= \frac{{(\sum_{i=1}^{k}{\lambda }_{ij})}^{2}}{{(\sum_{i=1}^{k}{\lambda }_{ij})}^{2}+ \sum_{i=1}^{k}{\varepsilon }_{ij}}$$

The threshold for reliability is 0.7 [41], which again was met for all factors. Table 17 summarizes the validity and reliability measures, as well as inter-factor correlations.

**Table 18:** Means, standard deviation, and quartiles

	M	SD	P25	P50	P75
CR	3.539	0.996	3.000	3.750	4.250
GR	3.274	1.176	2.000	3.600	4.200
KR	3.505	1.0819	2.666	3.666	4.333
DR	3.952	0.8516	3.500	4.000	4.750
BR	3.772	0.9084	3.000	4.000	4.500
Mission	3.392	1.0434	2.555	3.666	4.222
Total Score	3.655	0.8016	3.166	3.763	4.256

Finally, the last psychometric property to be considered is *sensitivity*, that is—the scale’s capability to differentiate between individuals. This requires that each individual item has a sufficiently normal distribution [68]. This is considered to be attained when the skewness and kurtosis are under the absolute value of 3 [57], which was already demonstrated in the EFA section. As such, all psychometric properties of the scale were fully demonstrated.

### Measurement invariance

In this step, we demonstrate measurement invariance for gender and persona experience—meaning that the scale is equally valid across those groups. To do this, a multi-group analysis was done using the procedure outlined by Marôco [68], in which the unconstrained model (i.e., the default one) is compared with models with increasing constraints. For this exercise, we contrast the unconstrained model with a model with fully constrained measurement weights and a model with fully constrained structural covariances. Differences are tested with chi-square tests, in which a non-significant result indicates measurement invariance.

We began by testing measurement invariance across persona experience. Since some of the response levels had few participants (e.g., “Novice” had 7), the variable was recorded so that “Novice” and “Beginner” were grouped as “Less Experienced” and “Experienced” and “Expert” as “More Experienced”. After comparing both groups, it was shown that the model with fully constrained measurement weights was not statistically different from the unconstrained model (χ^2^(13) = 7.633, *p* = 0.867), and the model with constrained structural covariances was likewise not statistically different (χ^2^(24) = 22,207, *p* = 0.567). As such, measurement invariance was demonstrated across levels of persona experience. We repeated this exercise, but for the male and female groups. Again, it was shown that the model with fully constrained measurement weights was not statistically different from the unconstrained model (χ^2^(13) = 12.889, *p* = 0.456), and the model with constrained structural covariances was also not statistically different (χ^2^(24) = 22,412, *p* = 0.555). As such, measurement invariance was demonstrated across both genders.

In conclusion, this exercise demonstrated that the scale can be used without needed changes regardless of the respondent’s gender and previous experience with personas.

Percentile analysis and norm creation.

For scoring purposes, Table 18 includes the means, standard deviations, and quartile cut-offs for our sample, using scores computed based on the final model. The discussion section includes guidelines based on these values.

**Table 19:** Correlations of PRS and its subscales with perceived success of persona projects

Variable	CR	GR	KR	DR	BR	MN	Total PR
Perceived Persona Success	0.512***	0.694***	0.702***	0.364***	0.454***	0.693***	0.722***

***p < 0.001; **p < 0.01; *p < 0.05

## Nomological validity

In order to assess nomological validity, we employed an additional question regarding the perceived success of the projects in the organization employing personas (“How successful has your persona project(s) been so far?”) using the EXPLORATORY SAMPLE. The response options were implemented using a semantic differential scale, ranging from Unsuccessful (1) to Successful (10). We correlated the score in this question with the composite means for all sub-dimensions of the PRS, as well as the global score. The correlations in Table 19 show that all dimensions of the PRS correlate strongly with perceived success, which we interpret as an indication of nomological validity as this is an expected correlation.

## Discussion and implications

### Theoretical implications

The use of personas has attracted researchers and practitioners from a variety of disciplines (e.g., computer science, ergonomics, HCI, UX/usability, psychology, and sociology), both in academia and industry. Despite this, systematic analysis of persona implementation and active use is missing from the current literature, with a major focus being on persona creation and application on isolated projects that report conflicting findings. Therefore, while there is much prior work focused on the personas as an instrument and on the use of personas for specific projects, there is, to our knowledge, little to no work focused on the organizational preparedness to actually employ personas.

While some prior studies report positive effects from persona use [18, 77, 93], others report negative [70, 88, 89] or neutral [35] effects. In this research, we proposed that organizational readiness could be a factor explaining these conflicting findings. Thus, future attention in the persona domain should be paid to the organization-wide implementation of personas, including education, investment, and employment [49]. To this end, the research reported here contributes by providing a measurement scale for organizational readiness for personas based on literature and is tested and validated using three independently collected samples. Our findings indicate that a persona project within an organization is a process that benefits from certain conditions for the implementation to be successful, specifically at three stages:

- *Readiness*, at the initial stage, occurs when the organization is receptive to and capable of managing the forthcoming persona project.
- *Adoption* would occur when the team members change their behaviors and attitudes to apply personas in their work.
- *Performance* occurs when personas become a stable part of employees’ behavior and fabric, positively affecting the level of user-centric decision making (e.g., creating more user-friendly products) and thereby providing positive performance outcomes for the organization.

As such, an area of future theoretical research is ‘organizational personas’ that can be used to assess and communicate about the company’s readiness to employ personas within individual projects or company initiatives [4]. One organization could have several personas, for example, to represent different departments or divisions within the organization. As such, organizational personas are an exciting area for future research and investigation.

### Practical implications

Organizational personas could be used to move the organization as a whole and specific divisions individually to persona readiness. However, there are general guidelines that seem reasonable. We propose that the PRS be deployed *before* moving to persona creation for specific projects; thus, the steps of an ideal persona project are as follows. *Persona readiness assessment *→* (Persona readiness improvement) *→* Persona creation *→* Persona deployment *→* Persona monitoring*. According to this logic, the chances of success with personas can be improved by assessing (and improving upon) the persona readiness of the organization using the above process.

Additionally, the PRS can be used to investigate persona readiness at multiple levels of examination: (a) how ready organizations are, *in general*, for personas; (b) how readiness differs by *industry or domain of application*; (c) how ready *a specific organization* is to launch a persona project; and (d) how ready *specific divisions within an organization* are to launch a persona project. Hence, the PRS provides a flexible and opportune starting point to systematically analyze persona readiness in the organizational sphere.

Using the scale also provides commercial opportunities for service organizations. For example, design consultancies offering persona creation and training services can use the PRS, along with the suggested guidelines for interpreting the results, to improve their clients’ persona readiness before launching costly persona projects. The PRS can help identify specific areas of improvement. Based on the results, tailored recommendations can be given to an organization. Therefore, we encourage decision-makers and persona champions within organizations to apply the PRS to gain valuable understanding of an organization’s general propensity for a persona project. Based on the results, decision-makers can develop realistic expectations and goals for persona use and develop a supportive climate for personas. For example, say that an organization ranks relatively high on other dimensions except on goal readiness. A further examination reveals that the scores for a plan for deployment and metrics (GR01, GR04) are especially low. The organization now directly knows to address these shortcomings to increase their persona readiness.

There are multiple methods for PRS deployment. The data from the PRS can be used to get help to create an organizational persona, which will aid in the creation of measures to get the organization persona ready. The PRS can be deployed using a standard Likert Scale, e.g., ranging from Strongly Disagree (1) to Strongly Agree (5). To interpret the results of a given organization, we recommend using the cutoff quartiles reported in Table 18. Applying the quartiles across three classes, we obtain the following interpretation of scores:

- A mean score of 3.17 and below indicates *Low Persona Readiness*
- A mean score between 3.18−4.25 indicates *Medium Persona Readiness*
- A mean score of 4.26 and above indicates *High Persona Readiness*

This straightforward scoring scheme has two advantages: (a) its computation is easy, and (b) it is based on benchmark data on more than 300 organizations. In contrast, a more advanced scoring scheme can be applied by assigning weights based on more and less essential dimensions for a given use case (e.g., when data requirements are seen as less stringent due to applying qualitative persona creation). Guidelines for exact sample sizes are difficult to give, and the number of people taking the PRS depends on the organization’s size.

We advise deploying the scale at multiple levels of the organization (both breadth and depth), asking multiple people in different departments and job roles (again, both breadth and depth) to complete the survey before making assessments of an organization’s readiness. When multiple people in the organization complete the PRS, the scores will be assigned based on the average ratings given by all the respondents.

Finally, implementing personas in organizations may require substantial organizational change – which often requires education about personas, their use, and their advantages. Increasing an organization’s persona readiness is not a trivial task. Therefore, it may take considerable effort to improve persona readiness and overcome elements of friction and resistance [103], such as perceiving personas as an irrelevant tool [70], lacking management support, and creating a supportive culture [103]. This means that following up on the survey results is crucial – again, requiring education to change negative perceptions and overcome resistance. Thus, PRS is the starting point for more work on improving organizational conditions for successful persona adoption.

### Future research directions

Further research is needed to fully understand the properties of the PRS. First, the test–retest reliability of the scale could be evaluated by repeating the test with the same respondents at different times. Future investigations using the scale could test for mediators or moderators, such as trust [101] and cross-cultural factors [80] that affect the impact of persona readiness. Second, the PRS was designed to apply to all kinds of personas, including those created using qualitative, quantitative, and mixed methods [48]. Nonetheless, the requirements for readiness slightly vary according to the persona creation methodology. If an organization decides to pursue quantitative personas created using algorithms [5, 6], they face additional data science competencies and resources requirements. Hence, it might be possible to assign different weights for the items based on the resources and capabilities that the organization requires for its specific persona project. However, doing so requires further investigation, which we leave for future research.

Third, it would be highly interesting to investigate whether organizational readiness for personas varies by industry. If the PRS were to be deployed broadly across different fields such as manufacturing, UX, marketing, software, and so on, it could help create favorable conditions for persona projects in multiple fields. Finally, future studies will also need to look at the PRS in action. As reflected in the scale, personas need advocacy, and future studies of interest could be to see which job roles instigate the use of the PRS and if this creates an impact, alongside investigating if the scale is used once or applied at regular basis to determine improvements. These explorations remain crucial directions for future research, especially how well persona readiness predicts performance outcomes, in terms of the design quality of products and the achievement of organizational goals in general.

## Conclusion

In this work, we proposed a persona readiness scale. The validated scale has five dimensions and eighteen items, and it accommodates qualitative, quantitative, and mixed-method personas. Organizations can administer the scale directly or with the help of design agencies before committing to expensive persona projects. Knowing the current state of persona readiness can help the organization locate points of improvement. As persona creation is costly, time-consuming, and resource-intensive, any activities that may improve the compatibility between the organization and personas should be undertaken when aiming at successful persona projects incorporating user-centric design thinking in the development of IT products.

## Supplementary Information

Below is the link to the electronic supplementary material.

Supplementary file 1 (DOCX 60 kb)

## Footnotes

1 See Appendix 1, Table 20.

2 See Appendix 1, Table 21 and 22. In addition to the Appendix included with this article, the data in spreadsheet form is available at: https://www.dropbox.com/s/adsrhjgdzfaljfc/APPENDIX_1_PRS.xlsx?dl=0.

3 See Appendix 1, Table 23.

4 See Appendix 1, Table 22.

5 See Appendix 1, Table 23.

6 https://www.dropbox.com/s/n3g7kl80p24mpo7/APPENDIX%202_PRS.xlsx?dl=0.

7 Obtained from https://www.gov.uk/national-minimum-wage-rates (April, 2021).

8 Although it was not reported here for the sake of parsimony, an EFA-based route was attempted at the CFA stage, prior to using the strategy which is reported in the next section. The EFA-based structure ended up performing poorly at the CFA stage and was inferior to the solution which was obtained through the exploratory CFA strategy.

9 These concepts regarding validity will be explained in greater depth further ahead, in the section which is dedicated to discriminant and convergent validity.

## References

[1] Abdi H (2003). Factor rotations in factor analyses. Encyclopedia for Research Methods for the Social Sciences. Sage, Thousand Oaks.

[2] Adlin T, Pruitt J (2010). The essential persona lifecycle: your guide to building and using personas.

[3] Aimé I, Berger-Remy F, Laporte M-E (2022). The brand, the persona and the algorithm: how datafication is reconfiguring marketing work☆. J Bus Res.

[4] Ali F, Stewart R, Boks C, Bey N (2019). Exploring “Company Personas” for informing design for sustainability implementation in companies. Sustainability.

[5] An J, Kwak H, Jung S-G, Salminen J, Jansen BJ (2018). Customer segmentation using online platforms: isolating behavioral and demographic segments for persona creation via aggregated user data. Soc Netw Anal Min.

[6] An J, Kwak H, Salminen J, Jung S-G, Jansen BJ (2018). Imaginary people representing real numbers: generating personas from online social media data. ACM Trans Web (TWEB).

[7] Anderson DR, Burnham KP, White GC (1998). Comparison of Akaike information criterion and consistent Akaike information criterion for model selection and statistical inference from capture-recapture studies. J Appl Stat.

[8] Anvari F, Richards D (2016) A method to identify talented aspiring designers in use of personas with personality. In: Maciaszek LA, Filipe J (eds) Evaluation of novel approaches to software engineering. Springer, Cham, pp 40–61. 10.1007/978-3-319-30243-0_3

[9] Anvari F, Richards D, Hitchens M, Tran HMT (2019) Teaching user centered conceptual design using cross-cultural personas and peer reviews for a large cohort of students. In: 2019 IEEE/ACM 41st international conference on software engineering: software engineering education and training (ICSE-SEET), pp 62–73. 10.1109/ICSE-SEET.2019.00015

[10] Anvari F, Tran HMT (2013) Persona ontology for user centred design professionals. In: The ICIME 4th international conference on information management and evaluation, Ho Chi Minh City, Vietnam, pp 35–44

[11] Arbuckle J (2007). Amos 160 user’s guide.

[12] Arkush ES, Stanton SA (1988). Measuring the value of end-user computing. J Inf Syst Manag.

[13] Arnold KE, Lonn S, Pistilli MD (2014) An exercise in institutional reflection: the learning analytics readiness instrument (LARI). In: Proceedings of the fourth international conference on learning analytics and knowledge, pp 163–167

[14] Barrett P (2007). Structural equation modelling: Adjudging model fit. Person Individ Differ.

[15] Bentler PM (1990). Comparative fit indexes in structural models. Psychol Bull.

[16] Bentler PM (2007). On tests and indices for evaluating structural models. Person Individ Differ.

[17] Billestrup J, Stage J, Bruun A, Nielsen L, Nielsen KS (2014) Creating and using personas in software development: experiences from practice. In: Human-centered software engineering (Lecture Notes in Computer Science), Springer, Berlin, Heidelberg, pp 251–258. 10.1007/978-3-662-44811-3_16

[18] Blomquist A, Arvola M (2002) Personas in action: ethnography in an interaction design team. In: Proceedings of the second Nordic conference on human–computer interaction, pp 197–200

[19] Bødker S, Christiansen E, Nyvang T, Zander P-O (2012) Personas, people and participation: challenges from the trenches of local government. In: Proceedings of the 12th participatory design conference on research papers: volume 1 - PDC ’12, ACM Press, Roskilde, Denmark, p 91. 10.1145/2347635.2347649

[20] Bollen KA (2014). Structural equations with latent variables.

[21] Brauner P, Philipsen R, Valdez AC, Ziefle M (2019). What happens when decision support systems fail? The importance of usability on performance in erroneous systems. Behav Inform Technol.

[22] Carroll JM (1997) Scenario-based design. In: Handbook of human–computer interaction. Elsevier, pp 383–406

[23] Chapman C, Love E, Milham RP, ElRif P, Alford JL (2008). Quantitative Evaluation of Personas as Information. Proc Human Factors Ergonom Soc Annu Meet.

[24] Chapman C, Milham RP (2006). The Personas’ new clothes: methodological and practical arguments against a popular method. Proc Human Factors Ergonom Soc Annu Meet.

[25] Chapman L, Plewes S (2014) A UX maturity model: effective introduction of UX into organizations. In: International conference of design, user experience, and usability. Springer, Cham, pp 12–22

[26] Cooper A (1999). The Inmates Are Running the Asylum: Why High Tech Products Drive Us Crazy and How to Restore the Sanity.

[27] Dinda PA, Memik G, Dick RP, Lin B, Mallik A, Gupta A, Rossoff S (2007) The user in experimental computer systems research. In: Proceedings of the 2007 workshop on experimental computer science, 10-es

[28] DiStefano C, Min Zhu, Diana Mindrila (2009) Understanding and using factor scores: considerations for the applied researcher. Practical Assessment, Research & Evaluation 14(20):1–11

[29] Ellemieke Van Doorn, Rusák Z, Horváth I (2017) A situation awareness analysis scheme to identify deficiencies of complex man-machine interactions. Int J Inform Technol Manag 16(1):53–72

[30] Ferreira BM, Barbosa SDJ, Conte T ( 2016) PATHY: using empathy with personas to design applications that meet the users’ needs. In: Human-computer interaction. theory, design, development and practice (Lecture Notes in Computer Science). Springer International Publishing, Cham, pp 153–165. 10.1007/978-3-319-39510-4_15

[31] Fonteyn ME, Kuipers B, Grobe SJ (2016) A description of think aloud method and protocol analysis: qualitative health research. 10.1177/104973239300300403

[32] Fornell C, Larcker DF (1981). Evaluating structural equation models with unobservable variables and measurement error. J Mark Res.

[33] Fraser J, Plewes S (2015). Applications of a UX maturity model to influencing HF best practices in technology centric companies–Lessons from Edison. Procedia Manuf.

[34] Frich J, Biskjaer MM, Remy C, Vermulen LM, Dalsgaard P (2021). User research and design creativity: three insights for future studies. Behav Inform Technol.

[35] Friess E (2012). Personas and decision making in the design process: an ethnographic case study. In: Proceedings of the SIGCHI conference on human factors in computing systems.

[36] Goh CH, Kulathuramaiyer N, Zaman T (2017) Riding waves of change: a review of personas research landscape based on the three waves of HCI. In: Information and communication technologies for development (IFIP Advances in Information and Communication Technology), Springer, Cham, pp 605–616. 10.1007/978-3-319-59111-7_49

[37] Grudin J (2006) Why personas work: the psychological evidence. In: The persona lifecycle. In: Pruitt J, Adlin T (eds) Elsevier, pp 642–663. 10.1016/B978-012566251-2/50013-7

[38] Guðjónsdóttir R, Lindquist S (2008) Personas and scenarios: design tool or a communication device. In: 8th International conference on cooperative systems (COOP’08), pp 165–176

[39] Gummesson E (2006). Qualitative research in management: addressing complexity, context and persona. Manag Decis.

[40] Haag M, Marsden N (2019). Exploring personas as a method to foster empathy in student IT design teams. Int J Technol Design Educ.

[41] Hair JF, Black WC, Babin BJ, Anderson RE (2014). Multivariate data analysis.

[42] Howard TW (2015). Are personas really usable?. Commun Design Q Rev.

[43] Li-tze Hu, Bentler PM (1999). Cutoff criteria for fit indexes in covariance structure analysis: Conventional criteria versus new alternatives. Struct Equ Model: Multidiscip J.

[44] Huang X, Lei Wu, Ye Y (2019). A review on dimensionality reduction techniques. Int J Pattern Recognit Artif Intell.

[45] Hung S-Y, Hung W-H, Tsai C-A, Jiang S-C (2010). Critical factors of hospital adoption on CRM system: organizational and information system perspectives. Decision Support Syst.

[46] Idoughi D, Seffah A, Kolski C (2012). Adding user experience into the interactive service design loop: a persona-based approach. Behav Inform Technol.

[47] Jansen BJ, Salminen J, Jung S-G (2020). Data-driven personas for enhanced user understanding: combining empathy with rationality for better insights to analytics. Data Inform Manag.

[48] Jansen B, Jung S-G, Nielsen L, Guan KW, Salminen J (2022). How to create personas: three persona creation methodologies with implications for practical employment. Pacific Asia J Assoc Inform Syst.

[49] Jansen B, Salminen J, Jung S, Guan K (2021) Data-Driven Personas (1st ed.). Morgan & Claypool Publishers

[50] Jensen I, Hautopp H, Nielsen L, Madsen S (2017) Developing international personas: A new intercultural communication practice in globalized societies. J Intercultural Commun 43

[51] Jung S-G, Salminen J, An J, Kwak H, Jansen BJ (2018) Automatically conceptualizing social media analytics data via personas. In: Proceedings of the international AAAI conference on Web and Social Media (ICWSM 2018), San Francisco, California, USA, 2

[52] Jung S, Salminen J, Jansen BJ (2019) Personas changing over time: analyzing variations of data-driven personas during a two-year period. In: Extended abstracts of the 2019 CHI conference on human factors in computing systems - CHI EA ’19, ACM Press, Glasgow, Scotland, pp 1–6. 10.1145/3290607.3312955

[53] Jung S-G, Salminen J, Jansen BJ (2020) Giving faces to data: creating data-driven personas from personified Big Data. In: Proceedings of the 25th international conference on intelligent user interfaces companion (IUI ’20), Association for Computing Machinery, Cagliari, Italy, pp 132–133. 10.1145/3379336.3381465

[54] Jung S, Salminen J, Kwak H, An J, Jansen BJ (2018) Automatic Persona Generation (APG): A rationale and demonstration. In: CHIIR ’18: Proceedings of the 2018 conference on human information interaction & retrieval. ACM, New Jersey, USA, pp 321–324. 10.1145/3176349.3176893

[55] Kankainen A, Vaajakallio K, Kantola V, Mattelmäki T (2012). Storytelling Group–a co-design method for service design. Behaviour & Information Technology.

[56] Katzeff C, Nyblom Å, Tunheden S, Torstensson C (2012). User-centred design and evaluation of EnergyCoach–an interactive energy service for households. Behaviour & Information Technology.

[57] Kline RB (2016). Principles and practice of structural equation modeling.

[58] Kolenikov S, Bollen KA (2012). Testing Negative Error Variances Is a Heywood Case a Symptom of Misspecification?. Sociological Methods & Research.

[59] Li H, Chen Qi, Zhong Z, Gong R, Han G (2022). E-word of mouth sentiment analysis for user behavior studies. Inf Process Manage.

[60] Li Y, Yuan X, Che R (2021). An investigation of task characteristics and users’ evaluation of interaction design in different online health information systems. Inf Process Manage.

[61] Lim SAH, Antony J (2016). Statistical process control readiness in the food industry: Development of a self-assessment tool. Trends Food Sci Technol.

[62] Lin C, Kunnathur A (2019). Strategic orientations, developmental culture, and big data capability. J Bus Res.

[63] Liu C, Liu Y-H, Liu J, Bierig R (2021). Search interface design and evaluation. INR.

[64] Lokuge S, Sedera D, Grover V, Dongming Xu (2019). Organizational readiness for digital innovation: Development and empirical calibration of a construct. Information & management.

[65] Long F (2009) Real or imaginary: the effectiveness of using personas in product design. In: Proceedings of the Irish Ergonomics Society Annual Conference, Irish Ergonomics Society Dublin

[66] Marcus A, Gunther R, Sieffert R (2009) Validating a standardized usability/user-experience maturity model: a progress report. In: International conference on human centered design. Springer, pp 104–109

[67] Maroco J (2003) Análise estatística: com utilização do SPSS

[68] Marôco J (2010) Análise de equações estruturais: Fundamentos teóricos, software & aplicações. ReportNumber, Lda

[69] Marsden N, Pröbster M, Ehsanul Haque M, Hermann J (2017) Cognitive styles and personas: designing for users who are different from me. In: Proceedings of the 29th Australian conference on computer-human interaction. ACM, Brisbane, Queensland, Australia, pp 452–456

[70] Matthews T, Judge T, Whittaker S (2012) How do designers and user experience professionals actually perceive and use personas? In: Proceedings of the 2012 ACM annual conference on human factors in computing systems - CHI ’12. ACM Press, Austin, Texas, USA, p 1219. 10.1145/2207676.2208573

[71] Matthews T, Whittaker S, Moran T, Yuen S (2011) Collaboration personas: a new approach to designing workplace collaboration tools. In: Proceedings of the SIGCHI conference on human factors in computing systems, pp 2247–2256

[72] McGinn JJ, Kotamraju N (2008) Data-driven persona development. In: Proceedings of the SIGCHI conference on human factors in computing systems. ACM, Florence, Italy. 10.1145/1357054.1357292

[73] Molich R, Dumas JS (2008). Comparative usability evaluation (CUE-4). Behaviour & Information Technology.

[74] Mulder S, Yaar Z (2006) The user is always right: a practical guide to creating and using personas for the Web. New Riders

[75] Nielsen L (2019) Personas - user focused design (2nd ed. 2019 edition ed.). Springer, New York

[76] Nielsen L, Hansen KS, Stage J, Billestrup J (2015). A template for design personas: analysis of 47 persona descriptions from danish industries and organizations. Int J Sociotechnol Knowl Develop.

[77] Nielsen L, Storgaard Hansen K (2014) Personas is applicable: a study on the use of personas in Denmark. In: Proceedings of the SIGCHI conference on human factors in computing systems. ACM, Toronto, pp 1665–1674

[78] Nieters JE, Ivaturi S, Ahmed I (2007) Making personas memorable. In: CHI ’07 extended abstracts on Human factors in computing systems - CHI ’07. ACM Press, San Jose, p 1817. 10.1145/1240866.1240905

[79] Orcan F (2018) Exploratory and confirmatory factor analysis: which one to use first? Eğitimde ve Psikolojide Ölçme ve Değerlendirme Dergisi (December 2018), pp 414–421. 10.21031/epod.394323

[80] Oshlyansky L, Cairns P, Thimbleby H (2007) Validating the Unified Theory of Acceptance and Use of Technology (UTAUT) tool cross-culturally. In: Proceedings of HCI 2007 the 21st British HCI Group Annual Conference University of Lancaster, UK 21, pp 1–4

[81] Øvad T, Larsen LB (2016) How to reduce the UX bottleneck–train your software developers. Behav Inform Technol 35(12):1080–1090

[82] Palan S, Schitter C (2018). Prolific. ac—a subject pool for online experiments. J Behav Exp Financ.

[83] Peer E, Brandimarte L, Samat S, Acquisti A (2017). Beyond the Turk: alternative platforms for crowdsourcing behavioral research. J Exp Soc Psychol.

[84] Pruitt J, Grudin J (2003) Personas: practice and theory. In: Proceedings of the 2003 conference on designing for user experiences (DUX ’03). ACM, San Francisco, California, USA, 1–15. 10.1145/997078.997089

[85] de Queiroz Tourinho A, Sanchez OP, Brown SA (2019) Measuring the organizational analytical competence: dDevelopment of a Scale. In: Proceedings of the 27th European Conference on Information Systems (ECIS), Stockholm & Uppsala, Sweden

[86] Ramaseshan B, Philip Kingshott R, Stein A (2015) Firm self-service technology readiness. J Serv Manag

[87] Robey D, Welke R, Turk D (2001). Traditional, iterative, and component-based development: a social analysis of software development paradigms. Inf Technol Manage.

[88] Rönkkö K (2005) An empirical study demonstrating how different design constraints, Project Organization and Contexts Limited the Utility of Personas. In: Proceedings of the proceedings of the 38th annual Hawaii international conference on system sciences - volume 08 (HICSS ’05). IEEE Computer Society, Washington, DC, USA. 10.1109/HICSS.2005.85

[89] Rönkkö K, Hellman M, Kilander B, Dittrich Y (2004) Personas is not applicable: local remedies interpreted in a wider context. In: Proceedings of the eighth conference on participatory design: artful integration: interweaving media, materials and practices - volume 1 (PDC 04). ACM, Toronto, pp 112–120. 10.1145/1011870.1011884

[90] Salminen J, Guan K, Jung S, Absar Chowdhury S, Jansen BJ (2020) A literature review of quantitative persona creation. In: CHI ’20: proceedings of the 2020 CHI conference on human factors in computing systems. ACM, Honolulu, Hawaii, USA, pp 1–14. 10.1145/3313831.3376502

[91] Salminen J, Guan K, Nielsen L, Soon-gyo Jung, Shammur Absar Chowdhury, and Bernard J. Jansen (2020) A template for data-driven personas: analyzing 31 quantitatively oriented persona profiles. In: Yamamoto S, Mori H (eds) Human interface and the management of information. designing information. HCII 2020. Springer, Copenhagen, pp 125–144

[92] Salminen J, Jansen BJ, An J, Kwak H, Jung S-G (2018). Are personas done? Evaluating their usefulness in the age of digital analytics. Persona Stud.

[93] Joni Salminen, Soon-gyo Jung, Shammur Absar Chowdhury, Sercan Sengün, and Bernard J Jansen. 2020. Personas and Analytics: A Comparative User Study of Efficiency and Effectiveness for a User Identification Task. In Proceedings of the ACM Conference of Human Factors in Computing Systems (CHI’20), ACM, Honolulu, Hawaii, USA. 10.1145/3313831.3376770

[94] Joni Salminen, Soon-gyo Jung, Ahmed Mohamed Sayed Kamel, João M. Santos, and Bernard J. Jansen. 2020. Using artificially generated pictures in customer-facing systems: an evaluation study with data-driven personas. Behaviour & Information Technology 0, 0 (November 2020), 1–17. 10.1080/0144929X.2020.1838610

[95] Salminen J, Jung S, Santos JM, Chowdhury S, Jansen BJ (2020) The effect of experience on persona perceptions. In: Extended abstracts of the 2020 CHI conference on human factors in computing systems extended abstracts (CHI ’20), Association for Computing Machinery, Honolulu, HI, USA, 1–9. 10.1145/3334480.3382786

[96] Salminen J, Jung S, Santos JM, Jansen BJ (2019) Does a smile matter if the person is not real?: The effect of a smile and stock photos on persona perceptions. Int J Hum–Comput Interact. 10.1080/10447318.2019.1664068

[97] Salminen J, Kaate I, Mohamed Sayed Kamel A, Jung S, Jansen BJ (2020) How does personification impact Ad performance and empathy? An experiment with online advertising. Int J Hum-Comput Interact. 10.1080/10447318.2020.1809246

[98] Salminen J, Nielsen L, Jung S-G, Jansen BJ (2021) Towards a measurement scale of organizational readiness for personas. In: Extended abstracts of CHI conference on human factors in computing systems (CHI2021), ACM, Virtual conference

[99] Salminen J, Santos JM, Jung S, Eslami M, Jansen BJ (2019). Persona transparency: analyzing the impact of explanations on perceptions of data-driven personas. Int J Hum-Comput Interact.

[100] Salminen J, Santos JM, Kwak H, An J, Jung S-G, Jansen BJ (2020). Persona Perception Scale: development and exploratory validation of an instrument for evaluating individuals’ perceptions of personas. Int J Hum-Comput Stud.

[101] Sanchez O, Brown S, Zhang B (2020) Trust and distrust in big data recommendation agents. In: 40th international conference on information systems, ICIS 2019, Association for Information Systems

[102] Sauro J, Johnson K, Meenan C (2017) From snake-oil to science: measuring UX maturity. In: Proceedings of the 2017 CHI conference extended abstracts on human factors in computing systems, pp 1084–1091

[103] Seidelin C, Jonsson A, Høgild M, Rømer J, Diekmann P (2014) Implementing personas for international markets: a question of UX maturity. In: Proceedings at SIDER

[104] Steiger JH, Shapiro A, Browne MW (1985). On the multivariate asymptotic distribution of sequential chi-square statistics. Psychometrika.

[105] Stevenson PD, Mattson CA (2019). The personification of Big Data. Proceedings of the design society: international conference on engineering design.

[106] Takai S, Ishii K (2010). A use of subjective clustering to support affinity diagram results in customer needs analysis. Concurr Eng.

[107] Tan H, Peng S, Liu J-X, Zhu C-P, Zhou F (2021). Generating personas for products on social media: a mixed method to analyze online users. Int J Hum-Comput Interact.

[108] Temkin BD (2008) The customer experience journey. Forrester Res

[109] Venkatesh V, Brown SA, Sullivan YW (2016). Guidelines for conducting mixed-methods research: an extension and illustration. J Assoc Inform Syst.

[110] Venkatesh V, Thong JYL, Xin Xu (2012). Consumer acceptance and use of information technology: extending the unified theory of acceptance and use of technology. MIS Q.

[111] Viana G, Robert J-M (2016) The practitioners’ points of view on the creation and use of personas for user interface design. In: International conference on human–computer interaction. Springer, pp 233–244

[112] Wang W, Guo L, Wu YJ, Goh M, Wang S (2022). Content-oriented or persona-oriented? A text analytics of endorsement strategies on public willingness to participate in citizen science. Inform Process Manag.

[113] Wang X, Guo Y, Yang M, Chen Y, Zhang W (2017). Information ecology research: past, present, and future. Inform Technol Manag.

[114] Worthington RL, Whittaker TA (2006). Scale development research: a content analysis and recommendations for best practices. Counsel Psychol.

[115] Zhang P (2003). Multiple imputation: theory and method. Int Stat Rev.

[116] Zhang P, Dillon A (2003). HCI and MIS: shared concerns. Int J Hum-Comput Stud.

[117] Zhang Y, Sun J, Yang Z, Wang Y (2020). Critical success factors of green innovation: technology, organization and environment readiness. J Clean Prod.

