# Supplementary material for: Persona preparedness: a survey instrument for measuring the organizational readiness for deploying personas
Source: Inf Technol Manag. 2022 Sep 13:1–26. Online ahead of print. doi: 10.1007/s10799-022-00373-9 (PMC9469059; doi:10.1007/s10799-022-00373-9)
Supplement: Supplementary file 1 — (DOCX 60 kb) [file 10799_2022_373_MOESM1_ESM.docx]

**Appendix 1 Tables for Literature Review**

**Table 20:** Articles Reviewed for Possible Indicators of Organizational Persona Readiness.

| **Article** |
| --- |
| Tamara Adlin and John Pruitt. 2010. The Essential Persona Lifecycle: Your Guide to Building and Using Personas (1st ed.). Morgan Kaufmann Publishers Inc., San Francisco, CA, USA. |
| Isabelle Aimé, Fabienne Berger-Remy, and Marie-Eve Laporte. 2022. The brand, the persona and the algorithm: How datafication is reconfiguring marketing work☆. Journal of Business Research 145, (June 2022), 814–827. DOI:https://doi.org/10.1016/j.jbusres.2022.03.047 |
| Faheem Ali, Raphaëlle Stewart, Casper Boks, and Niki Bey. 2019. Exploring “Company Personas” for Informing Design for Sustainability Implementation in Companies. Sustainability 11, 2 (January 2019), 463. DOI:https://doi.org/10.3390/su11020463 |
| Farshid Anvari and Deborah Richards. 2016. A Method to Identify Talented Aspiring Designers in Use of Personas with Personality. In Evaluation of Novel Approaches to Software Engineering, Leszek A. Maciaszek and Joaquim Filipe (eds.). Springer International Publishing, Cham, 40–61. DOI:https://doi.org/10.1007/978-3-319-30243-0_3 |
| Farshid Anvari, Deborah Richards, Michael Hitchens, and Hien Minh Thi Tran. 2019. Teaching User Centered Conceptual Design Using Cross-Cultural Personas and Peer Reviews for a Large Cohort of Students. In 2019 IEEE/ACM 41st International Conference on Software Engineering: Software Engineering Education and Training (ICSE-SEET), 62–73. DOI:https://doi.org/10.1109/ICSE-SEET.2019.00015 |
| Farshid Anvari and Hien Minh Tri Tran. 2013. Persona ontology for user centred design professionals. In The ICIME 4th International Conference on Information Management and Evaluation, Ho Chi Minh City, Vietnam, 35–44. |
| Jane Billestrup, Jan Stage, Anders Bruun, Lene Nielsen, and Kira S. Nielsen. 2014. Creating and Using Personas in Software Development: Experiences from Practice. In Human-Centered Software Engineering (Lecture Notes in Computer Science), Springer, Berlin, Heidelberg, 251–258. DOI:https://doi.org/10.1007/978-3-662-44811-3_16 |
| Asa Blomquist and Mattias Arvola. 2002. Personas in action: ethnography in an interaction design team. In Proceedings of the second Nordic conference on Human-computer interaction, 197–200. |
| Susanne Bødker, Ellen Christiansen, Tom Nyvang, and Pär-Ola Zander. 2012. Personas, people and participation: challenges from the trenches of local government. In Proceedings of the 12th Participatory Design Conference on Research Papers: Volume 1 - PDC ’12, ACM Press, Roskilde, Denmark, 91. DOI:https://doi.org/10.1145/2347635.2347649 |
| Chris Chapman, Edwin Love, Russell P. Milham, Paul ElRif, and James L. Alford. 2008. Quantitative Evaluation of Personas as Information. In Proceedings of the Human Factors and Ergonomics Society Annual Meeting, 1107–1111. DOI:https://doi.org/10.1177/154193120805201602 |
| Chris Chapman and Russell P. Milham. 2006. The Personas’ New Clothes: Methodological and Practical Arguments against a Popular Method. In Proceedings of the Human Factors and Ergonomics Society Annual Meeting, 634–636. DOI:https://doi.org/10.1177/154193120605000503 |
| Alan Cooper. 1999. The Inmates Are Running the Asylum: Why High Tech Products Drive Us Crazy and How to Restore the Sanity (1 edition ed.). Sams - Pearson Education, Indianapolis, IN. |
| Bruna Moraes Ferreira, Simone D. J. Barbosa, and Tayana Conte. 2016. PATHY: Using Empathy with Personas to Design Applications that Meet the Users’ Needs. In Human-Computer Interaction. Theory, Design, Development and Practice (Lecture Notes in Computer Science), Springer International Publishing, Cham, 153–165. DOI:https://doi.org/10.1007/978-3-319-39510-4_15 |
| Erin Friess. 2012. Personas and decision making in the design process: an ethnographic case study. In Proceedings of the SIGCHI Conference on Human Factors in Computing Systems, 1209–1218. DOI:https://doi.org/10.1145/2207676.2208572 |
| Jonathan Grudin. 2006. Why Personas Work: The Psychological Evidence. In The Persona Lifecycle, John Pruitt and Tamara Adlin (eds.). Elsevier, 642–663. DOI:https://doi.org/10.1016/B978-012566251-2/50013-7 |
| Rósa Guðjónsdóttir and Sinna Lindquist. 2008. Personas and Scenarios: Design Tool or a Communication Device. In 8th International Conference on Cooperative Systems (COOP’08), 165–176. |
| Gummesson, E. 2006. Qualitative research in management: addressing complexity, context and persona. Management Decision 44, 2 (2006), 167–179. |
| Maren Haag and Nicola Marsden. 2019. Exploring personas as a method to foster empathy in student IT design teams. International Journal of Technology and Design Education 29, 3 (May 2019), 565–582. DOI:https://doi.org/10.1007/s10798-018-9452-5 |
| T. W. Howard. 2015. Are Personas Really Usable? Communication Design Quarterly Review 3, 2 (2015), 20–26. DOI:https://doi.org/10.1145/2752853.2752856 |
| Bernard J. Jansen, Joni Salminen, and Soon-gyo Jung. 2020. Data-Driven Personas for Enhanced User Understanding: Combining Empathy with Rationality for Better Insights to Analytics. Data and Information Management 4, 1 (2020), 1–17. DOI:https://doi.org/10.2478/dim-2020-0005 |
| Bernard Jansen, Soon-Gyo Jung, Lene Nielsen, Kathleen W. Guan, and Joni Salminen. 2022. How to Create Personas: Three Persona Creation Methodologies with Implications for Practical Employment. Pacific Asia Journal of the Association for Information Systems 14, 3 (2022). DOI:https://doi.org/10.17705/1pais.14301 |
| Bernard Jansen, Joni Salminen, Soon-gyo Jung, and Kathleen Guan. 2021. Data-Driven Personas (1st ed.). Morgan & Claypool Publishers. Retrieved February 10, 2021 from <https://www.morganclaypool.com/doi/abs/10.2200/S01072ED1V01Y202101HCI048> |
| Iben Jensen, Heidi Hautopp, Lene Nielsen, and Sabine Madsen. 2017. Developing international personas: A new intercultural communication practice in globalized societies. Journal of Intercultural Communication 43 (2017). |
| Frank Long. 2009. Real or imaginary: The effectiveness of using personas in product design. In Proceedings of the Irish Ergonomics Society Annual Conference, Irish Ergonomics Society Dublin. |
| Tara Matthews, Tejinder Judge, and Steve Whittaker. 2012. How do designers and user experience professionals actually perceive and use personas? In Proceedings of the 2012 ACM annual conference on Human Factors in Computing Systems - CHI ’12, ACM Press, Austin, Texas, USA, 1219. DOI:https://doi.org/10.1145/2207676.2208573 |
| Tara Matthews, Steve Whittaker, Thomas Moran, and Sandra Yuen. 2011. Collaboration personas: A new approach to designing workplace collaboration tools. In Proceedings of the SIGCHI conference on human factors in computing systems, 2247–2256. |
| Jennifer Jen McGinn and Nalini Kotamraju. 2008. Data-driven persona development. In Proceedings of the SIGCHI Conference on Human Factors in Computing Systems, ACM, Florence, Italy, 1521–1524. DOI:https://doi.org/10.1145/1357054.1357292 |
| Lene Nielsen. 2019. Personas - User Focused Design (2nd ed. 2019 edition ed.). Springer, New York, NY, USA. |
| Lene Nielsen, Kira Storgaard Hansen, Jan Stage, and Jane Billestrup. 2015. A Template for Design Personas: Analysis of 47 Persona Descriptions from Danish Industries and Organizations. International Journal of Sociotechnology and Knowledge Development 7, 1 (2015), 45–61. DOI:https://doi.org/10.4018/ijskd.2015010104 |
| Lene Nielsen and Kira Storgaard Hansen. 2014. Personas is applicable: a study on the use of personas in Denmark. In Proceedings of the SIGCHI Conference on Human Factors in Computing Systems, ACM, Toronto, Ontario, Canada, 1665–1674. |
| James E. Nieters, Subbarao Ivaturi, and Iftikhar Ahmed. 2007. Making personas memorable. In CHI ’07 extended abstracts on Human factors in computing systems - CHI ’07, ACM Press, San Jose, CA, USA, 1817. DOI:https://doi.org/10.1145/1240866.1240905 |
| John Pruitt and Jonathan Grudin. 2003. Personas: Practice and Theory. In Proceedings of the 2003 Conference on Designing for User Experiences (DUX ’03), ACM, San Francisco, California, USA, 1–15. DOI:https://doi.org/10.1145/997078.997089 |
| Kari Rönkkö, Mats Hellman, Britta Kilander, and Yvonne Dittrich. 2004. Personas is Not Applicable: Local Remedies Interpreted in a Wider Context. In Proceedings of the Eighth Conference on Participatory Design: Artful Integration: Interweaving Media, Materials and Practices - Volume 1 (PDC 04), ACM, Toronto, Ontario, Canada, 112–120. DOI:https://doi.org/10.1145/1011870.1011884 |
| Joni Salminen, Kathleen Guan, Soon-gyo Jung, Shammur Absar Chowdhury, and Bernard J. Jansen. 2020. A Literature Review of Quantitative Persona Creation. In CHI ’20: Proceedings of the 2020 CHI Conference on Human Factors in Computing Systems, ACM, Honolulu, Hawaii, USA, 1–14. DOI:https://doi.org/10.1145/3313831.3376502 |
| Joni Salminen, Kathleen Guan, Lene Nielsen, Soon-gyo Jung, Shammur Absar Chowdhury, and Bernard J. Jansen. 2020. A Template for Data-Driven Personas: Analyzing 31 Quantitatively Oriented Persona Profiles. In Human Interface and the Management of Information. Designing Information. HCII 2020., S. Yamamoto and H. Mori (eds.). Springer, Copenhagen, Denmark, 125–144. |
| Joni Salminen, Bernard J. Jansen, Jisun An, Haewoon Kwak, and Soon-gyo Jung. 2018. Are personas done? Evaluating their usefulness in the age of digital analytics. Persona Studies 4, 2 (November 2018), 47–65. DOI:https://doi.org/10.21153/psj2018vol4no2art737 |
| Cathrine Seidelin, A. Jonsson, M. Høgild, J. Rømer, and P. Diekmann. 2014. Implementing personas for international markets: a question of UX maturity. In Proceedings at SIDER. |
| Phillip Douglas Stevenson and Christopher Andrew Mattson. 2019. The Personification of Big Data. Proceedings of the Design Society: International Conference on Engineering Design 1, 1 (July 2019), 4019–4028. DOI:https://doi.org/10.1017/dsi.2019.409 |
| Hao Tan, Shenglan Peng, Jia-Xin Liu, Chun-Peng Zhu, and Fan Zhou. 2021. Generating Personas for Products on Social Media: A Mixed Method to Analyze Online Users. International Journal of Human–Computer Interaction 0, 0 (November 2021), 1–12. DOI:https://doi.org/10.1080/10447318.2021.1990520 |
| Gabriela Viana and Jean-Marc Robert. 2016. The practitioners’ points of view on the creation and use of personas for user interface design. In International Conference on Human-Computer Interaction, Springer, 233–244. |
| Wei Wang, Lihuan Guo, Yenchun Jim Wu, Mark Goh, and Shouyi Wang. 2022. Content-oriented or persona-oriented? A text analytics of endorsement strategies on public willingness to participate in citizen science. Information Processing & Management 59, 2 (March 2022), 102832. DOI:https://doi.org/10.1016/j.ipm.2021.102832 |
|  |

**Table 21:** Search Phrases, Number of Search Research from Google Scholar and Science Direct, and Results Considered.

| **Search phrase** | **Google Scholar Results** | **Science Direct Results** | **Results Considered** |
| --- | --- | --- | --- |
| +technology +maturity +scale | 1,130,000 | 55,567 | 230 |
| +technology +readiness +scale | 696,000 | 19, 896 | 142 |
| +technology +maturity +instrument | 327,000 | 24,291 | 150 |
| +technology +readiness +instrument | 305,000 | 9,698 | 145 |
| +analytics +maturity +scale | 43,200 | 29,179 | 145 |
| +analytics +readiness +scale | 30,500 | 10,306 | 145 |
| +analytics +maturity +instrument | 20,100 | 15,661 | 150 |
| +analytics +readiness +instrument | 14,800 | 5,224 | 145 |
| +"big data" +maturity +scale | 25,900 | 1,529 | 145 |
| +"big data" +readiness +scale | 19,600 | 736 | 145 |
| +"big data" +maturity +instrument | 8,200 | 520 | 145 |
| +"big data" +readiness +instrument | 6,770 | 269 | 145 |
| +"artificial intelligence" +maturity +scale | 37,800 | 2,368 | 145 |
| +"artificial intelligence" +readiness +scale | 26,000 | 1,093 | 145 |
| +"artificial intelligence" +maturity +instrument | 14,300 | 958 | 150 |
| +"artificial intelligence" +readiness +instrument | 12,400 | 486 | 140 |
| +"data science" +maturity +scale | 7,980 | 342 | 145 |
| +"data science" +readiness +scale | 4,800 | 156 | 145 |
| +"data science" +maturity +instrument | 2,280 | 137 | 145 |
| +"data science" +readiness +instrument | 1,680 | 62 | 132 |
|  |  |  |  |

**Table 22:** Results by Publication Year and Search Phrase, Selected for Inclusion or Not, and Reason for Exclusion.

| **Publication Year** | **Search Phrase** | **Selected (yes/no)** | **If No, Reason for Exclusion** | **Exclusion Category** |
| --- | --- | --- | --- | --- |
| 2000 | +technology +readiness +scale | no | focuses on consumers, not organizations | focuses on consumers, not organizations |
| 2008 | +technology +maturity +scale | no | no items | no items |
| 2010 | +technology +maturity +scale | no | not peer-reviewed | not peer-reviewed full paper |
| 2013 | +technology +maturity +scale | no | no items | no items |
| 2015 | +technology +maturity +scale | no | no items | no items |
| 1995 | +technology +readiness +scale | no | not relevant for topic | does not correspond to our readiness definition |
| 2009 | +technology +readiness +scale | no | lit review; no items | no items |
| 2011 | +technology +maturity +scale | no | no items | no items |
| 2017 | +technology +maturity +scale | no | no items | no items |
| 2012 | +technology +maturity +scale | no | no items | no items |
| 2016 | +analytics +readiness +scale | no | dissertation | not peer-reviewed full paper |
| 2017 | +analytics +readiness +scale | no | develops a conceptual model | no items |
| 2013 | +analytics +readiness +instrument | no | workshop | not peer-reviewed full paper |
| 2013 | +analytics +readiness +instrument | no | workshop | not peer-reviewed full paper |
| 2015 | +analytics +readiness +instrument | no | does not contain items | no items |
| 2016 | +"big data" +maturity +scale | no | not available for download | not available for download |
| 2014 | +"big data" +readiness +scale | no | conceptual, no items | no items |
| 2017 | +"artificial intelligence" +readiness +scale | no | master's thesis | not peer-reviewed full paper |
| 2015 | +analytics +readiness +scale | no | not available for download | not available for download |
| 2019 | +"big data" +maturity +scale +cfa +items | yes |  |  |
| 2019 | +"big data" +maturity +scale +cfa +items | yes |  |  |
| 2016 | +technology +maturity +scale | no | Exemplary items only | Exemplary items only |
| 2010 | +technology +maturity +scale | no | no items | no items |
| 2005 | +technology +readiness +scale | yes |  |  |
| 2006 | +technology +readiness +scale | no | Exemplary items only | Exemplary items only |
| 2002 | +technology +readiness +scale | no | no items | no items |
| 2020 | +technology +readiness +scale | yes |  |  |
| 2019 | +technology +readiness +scale | no | no items | no items |
| 2019 | +technology +readiness +scale | yes |  |  |
| 2018 | +technology +readiness +scale | no | develops a conceptual model | no items |
| 2016 | +technology +readiness +scale | yes |  |  |
| 2006 | '+technology +readiness +instrument | no | no items | no items |
| 2011 | '+technology +readiness +instrument | no | no items | no items |
| 2002 | '+technology +readiness +instrument | no | Review | no items |
| 2006 | '+technology +readiness +instrument | no | Exemplary items only | Exemplary items only |
| 2014 | '+technology +readiness +instrument | no | Identifying key indicators to measure e readiness | no items |
| 2014 | +analytics +maturity +scale | yes |  |  |
| 2016 | +analytics +readiness +scale | no | no items | no items |
| 2017 | +analytics +readiness +scale | no | no items | no items |
| 2012 | +analytics +readiness +scale | no | no items | no items |
| 2016 | +analytics +readiness +scale | no | no items | no items |
| 2015 | +analytics +readiness +scale | no | no items | no items |
| 2017 | +"big data" +maturity +scale | no | Framework | no items |
| 2018 | +"big data" +readiness +scale | no | Framework | no items |
| 2019 | +"big data" +readiness +instrument | no | Framework | no items |
| 2006 | +"artificial intelligence" +readiness +scale | no | conceptual, no items | no items |
| 2017 | +"data science" +readiness +instrument | no | no items | no items |
| 2006 | +analytics +maturity +scale | no | Framework | no items |
| 2010 | +analytics +maturity +scale | no | no items | no items |
| 2019 | +"big data" +maturity +scale +cfa +items | no | no items | no items |
| 2016 | +analytics +readiness +scale | no | Identifying factors | duplicate from same authors |
| 2012 | technology maturity scale | no | Proposing a research methodology | no items |
|  |  |  |  |  |

**Table 23:** Variable Type, Constructs Attributes, With Article Title and Year.

| **Persona Alteration** | **Changed** | **Instrument Name** | **Sub-Dimension** | **Article Title** | **Year** |
| --- | --- | --- | --- | --- | --- |
| Our firm constantly monitors our level of commitment to serving customer needs. | no changes | - | Customer Orientation (CUSO) | Strategic orientations, developmental culture, and big data capability | 2019 |
| Our firm measures customer satisfaction systematically and frequently. | no changes | - | Customer Orientation (CUSO) | Strategic orientations, developmental culture, and big data capability | 2019 |
| The policy of this firm has been to always consider the most up-to-date production technology available. | Is TO correlated with QPR? | - | Technological Orientation (TO) | Strategic orientations, developmental culture, and big data capability | 2019 |
| We have a long tradition and reputation in our industry of attempting to be first to try out new technologies. | Is TO correlated with QPR? | - | Technological Orientation (TO) | Strategic orientations, developmental culture, and big data capability | 2019 |
| Technological innovation is readily accepted in our program/project management. | Is TO correlated with QPR? | - | Technological Orientation (TO) | Strategic orientations, developmental culture, and big data capability | 2019 |
| Technological innovation based on research results is readily accepted in our firm. | Is TO correlated with QPR? | - | Technological Orientation (TO) | Strategic orientations, developmental culture, and big data capability | 2019 |
| Our new products are always at the state of the art of the technology. | Is TO correlated with QPR? | - | Technological Orientation (TO) | Strategic orientations, developmental culture, and big data capability | 2019 |
| We are able to identify sources of digital customer data that could be used for persona creation. |  | - | Big Data Capability (BDC) | Strategic orientations, developmental culture, and big data capability | 2019 |
| We are able to collect big data about customers to be used for persona creation. |  | - | Big Data Capability (BDC) | Strategic orientations, developmental culture, and big data capability | 2019 |
| We are able to store large volumes of data about customers. |  | - | Big Data Capability (BDC) | Strategic orientations, developmental culture, and big data capability | 2019 |
| We adopt state of the art technologies to process customer data. |  | - | Big Data Capability (BDC) | Strategic orientations, developmental culture, and big data capability | 2019 |
| We are good at data analytics which is mainly data mining and statistical analysis. |  | - | Big Data Capability (BDC) | Strategic orientations, developmental culture, and big data capability | 2019 |
| We are good at text analytics that deals with unstructured textual format data. |  | - | Big Data Capability (BDC) | Strategic orientations, developmental culture, and big data capability | 2019 |
| We are good at web analytics that deals with web sites. |  | - | Big Data Capability (BDC) | Strategic orientations, developmental culture, and big data capability | 2019 |
| We rely on personas to identify new business opportunities. |  | - | Big Data Capability (BDC) | Strategic orientations, developmental culture, and big data capability | 2019 |
| We rely on personas to develop new products. |  | - | Big Data Capability (BDC) | Strategic orientations, developmental culture, and big data capability | 2019 |
| We rely on personas to enhance our innovativeness. |  | - | Big Data Capability (BDC) | Strategic orientations, developmental culture, and big data capability | 2019 |
| We rely on personas to formulate our business strategy. |  | - | Big Data Capability (BDC) | Strategic orientations, developmental culture, and big data capability | 2019 |
| The people in my organization who engage in Business Intelligence and Analytics activities have skills to do statistical analysis |  | Analytical Competence (ACOMP) | Analytical Skills | MEASURING THE ORGANIZATIONAL ANALYTICAL COMPETENCE: DEVELOPMENT OF A SCALE | 2019 |
| The people in my organization who engage in Business Intelligence and Analytics activities have skills to use advance datamining tools |  | Analytical Competence (ACOMP) | Analytical Skills | MEASURING THE ORGANIZATIONAL ANALYTICAL COMPETENCE: DEVELOPMENT OF A SCALE | 2019 |
| The people in my organization who engage in Business Intelligence and Analytics activities have skills to use personas. |  | Analytical Competence (ACOMP) | Analytical Skills | MEASURING THE ORGANIZATIONAL ANALYTICAL COMPETENCE: DEVELOPMENT OF A SCALE | 2019 |
| The people in my organization who engage in Business Intelligence and Analytics activities have skills to create personas. |  | Analytical Competence (ACOMP) | IT & Data Skill | MEASURING THE ORGANIZATIONAL ANALYTICAL COMPETENCE: DEVELOPMENT OF A SCALE | 2019 |
|  |  | Analytical Competence (ACOMP) | IT & Data Skill | MEASURING THE ORGANIZATIONAL ANALYTICAL COMPETENCE: DEVELOPMENT OF A SCALE | 2019 |
| We use social media data for persona creation. |  | Analytical Competence (ACOMP) | IT & Data Skill | MEASURING THE ORGANIZATIONAL ANALYTICAL COMPETENCE: DEVELOPMENT OF A SCALE | 2019 |
| We use Web analytics data for persona creation. |  |  |  |  |  |
| We use CRM data for persona creation. |  |  |  |  |  |
| Management understand the need for the firm to know how to use personas. |  | Firm SST readiness | managerial acquiescence | Firm self-service technology readiness | 2016 |
| ANTECEDENT: managerial acquiesence leads to higher persona maturity level |  | Firm SST readiness | managerial acquiescence | Firm self-service technology readiness | 2016 |
| ANTECEDENT: managerial acquiesence leads to higher persona maturity level |  | Firm SST readiness | managerial acquiescence | Firm self-service technology readiness | 2016 |
| ANTECEDENT: managerial acquiesence leads to higher persona maturity level |  | Firm SST readiness | managerial acquiescence | Firm self-service technology readiness | 2016 |
| The use of personas has enabled customers to be more engaged with our firm |  | Firm SST readiness | customer alignment | Firm self-service technology readiness | 2016 |
| The use of personas has helped us reduce customer experience failures |  | Firm SST readiness | customer alignment | Firm self-service technology readiness | 2016 |
| Support is readily provided to employees who need help with personas |  | Firm SST readiness | customer alignment | Firm self-service technology readiness | 2016 |
| Employees are actively involved with persona creation. |  | Firm SST readiness | employee engagement | Firm self-service technology readiness | 2016 |
| Employees are actively involved with implementing personas into the firm’s operation. |  | Firm SST readiness | employee engagement | Firm self-service technology readiness | 2016 |
| We devote adequate resources to ensure our employees have well-developed persona competencies. |  | Firm SST readiness | employee engagement | Firm self-service technology readiness | 2016 |
| We conduct in-house employee training to gain necessary knowledge on using personas. |  | Firm SST readiness | employee engagement | Firm self-service technology readiness | 2016 |
| The use of personas is compatible with our decision making processes. |  | Firm SST readiness | channel integration | Firm self-service technology readiness | 2016 |
| The personas have helped us meet the needs and wants of customers. |  | Technology reediness | Technology compatibility (TC) | Critical success factors of green innovation: Technology, organization and environment readiness | 2020 |
| The adoption of personas has increased our job effectiveness. |  | Technology reediness | Relative Advantage (RA) | Critical success factors of green innovation: Technology, organization and environment readiness | 2020 |
| The use of personas has enhanced our product/service quality. |  | Technology reediness | Relative Advantage (RA) | Critical success factors of green innovation: Technology, organization and environment readiness | 2020 |
| The use of personas has positively impacted our sales. |  |  |  |  |  |
| Our organization pays attention to personas in daily operations. |  | Organization readiness | Environmental Concern (EC) | Critical success factors of green innovation: Technology, organization and environment readiness | 2020 |
| Our organization incorporates personas in corporate strategy. |  | Organization readiness | Environmental Concern (EC) | Critical success factors of green innovation: Technology, organization and environment readiness | 2020 |
| Our organization encourages employees to think creatively when using personas. |  | Organization readiness | Innovation Capability (IC) | Critical success factors of green innovation: Technology, organization and environment readiness | 2020 |
| Our organization provides managerial support at all levels for the use of personas. |  | Organization readiness | Innovation Capability (IC) | Critical success factors of green innovation: Technology, organization and environment readiness | 2020 |
| To facilitate the use of personas, our organization makes resources available as possible. |  | Organization readiness | Innovation Capability (IC) | Critical success factors of green innovation: Technology, organization and environment readiness | 2020 |
| The use of personas increases our organization's operational efficiency. |  | Firm Performance (FP) |  | Critical success factors of green innovation: Technology, organization and environment readiness | 2020 |
| The use of personas increases our organization's market share. |  | Firm Performance (FP) |  | Critical success factors of green innovation: Technology, organization and environment readiness | 2020 |
| The use of personas increases our organization's corporate profitability. |  | Firm Performance (FP) |  | Critical success factors of green innovation: Technology, organization and environment readiness | 2020 |
| Our organization outperforms competitors in operation/Product cost | Does PO positively affect CA? | Competitive Advantage (CA) |  | Critical success factors of green innovation: Technology, organization and environment readiness | 2020 |
| Our organization outperforms competitors in product/service quality | Does PO positively affect CA? | Competitive Advantage (CA) |  | Critical success factors of green innovation: Technology, organization and environment readiness | 2020 |
| Our organization outperforms competitors in research and development (R&D) | Does PO positively affect CA? | Competitive Advantage (CA) |  | Critical success factors of green innovation: Technology, organization and environment readiness | 2020 |
| Our organization outperforms competitors in management effectiveness | Does PO positively affect CA? | Competitive Advantage (CA) |  | Critical success factors of green innovation: Technology, organization and environment readiness | 2020 |
| My organization allocates adequate ﬁnancial resources necessary to develop high-quality personas. |  | Digital innovation readiness survey | Resource readiness (R) | Organizational readiness for digital innovation: Development and empirical calibration of a construct | 2019 |
| My organization allocates adequate human resources necessary to create and adopt personas. |  | Digital innovation readiness survey | Resource readiness (R) | Organizational readiness for digital innovation: Development and empirical calibration of a construct | 2019 |
| My organization allocates adequate IT infrastructure resources necessary to update the personas periodically. |  | Digital innovation readiness survey | Resource readiness (R) | Organizational readiness for digital innovation: Development and empirical calibration of a construct | 2019 |
| Customer data sources in my organization is/are stable, up-to-date, and reliable. |  | Digital innovation readiness survey | IT readiness (T) | Organizational readiness for digital innovation: Development and empirical calibration of a construct | 2019 |
| We have access to a range of new technologies like cloud, mobile, social media, and big data analytics available to create personas. |  | Digital innovation readiness survey | IT readiness (T) | Organizational readiness for digital innovation: Development and empirical calibration of a construct | 2019 |
| Our analytics systems are stable, up-to-date, and reliable to help persona creation. |  | Digital innovation readiness survey | IT readiness (T) | Organizational readiness for digital innovation: Development and empirical calibration of a construct | 2019 |
| Our staff members have the right atttitudes to adopt personas when making decisions about customers. |  | Digital innovation readiness survey | Innovation valance (Iv) | Organizational readiness for digital innovation: Development and empirical calibration of a construct | 2019 |
| Our staff members are motivated to use personas. |  | Digital innovation readiness survey | Innovation valance (Iv) | Organizational readiness for digital innovation: Development and empirical calibration of a construct | 2019 |
| Our staff members are empowered to use personas when making decisions about customers. |  | Digital innovation readiness survey | Innovation valance (Iv) | Organizational readiness for digital innovation: Development and empirical calibration of a construct | 2019 |
| Our staﬀ members have the appropriate knowledge (i.e., technical, business process, and organizational) to effectively use personas. |  | Digital innovation readiness survey | Cognitive readiness (Cg) | Organizational readiness for digital innovation: Development and empirical calibration of a construct | 2019 |
| Our staﬀ members have the appropriate skills to use personas. |  | Digital innovation readiness survey | Cognitive readiness (Cg) | Organizational readiness for digital innovation: Development and empirical calibration of a construct | 2019 |
| Our staff members find innovative ways to use personas. |  | Digital innovation readiness survey | Cognitive readiness (Cg) | Organizational readiness for digital innovation: Development and empirical calibration of a construct | 2019 |
| Our customer analytics systems (e.g., CRM, Web analytics) are well-equipped to support persona creation. |  | Digital innovation readiness survey | Global measures of organizational readiness for digital innovations (Global) | Organizational readiness for digital innovation: Development and empirical calibration of a construct | 2019 |
| Our organization is well-equipped to support the adoption of personas. |  | Digital innovation readiness survey | Global measures of organizational readiness for digital innovations (Global) | Organizational readiness for digital innovation: Development and empirical calibration of a construct | 2019 |
| We are good at using personas in the organization. |  | Digital innovation readiness survey | Innovation implementation eﬀectiveness (Innovation) | Organizational readiness for digital innovation: Development and empirical calibration of a construct | 2019 |
| We have used personas to develop new products and services. |  | Digital innovation readiness survey | Innovation implementation eﬀectiveness (Innovation) | Organizational readiness for digital innovation: Development and empirical calibration of a construct | 2019 |
| We are using personas to communicate about customers in our internal team meetings. |  |  |  |  |  |
| We are using personas to create marketing messages for specific target groups. |  |  |  |  |  |
| Top management is ready to adopt personas in the organization. |  | SPC readiness self assessment tool | Top management support | Statistical process control readiness in the food industry: Development of a self-assessment tool | 2016 |
| Top management understands its role to start implementing personas. |  | SPC readiness self assessment tool | Top management support | Statistical process control readiness in the food industry: Development of a self-assessment tool | 2016 |
| Top management supports persona creation actitivies. |  | SPC readiness self assessment tool | Top management support | Statistical process control readiness in the food industry: Development of a self-assessment tool | 2016 |
| Top management is visibly committed to persona use in the organization. |  | SPC readiness self assessment tool | Top management support | Statistical process control readiness in the food industry: Development of a self-assessment tool | 2016 |
| Employeed are trained to use personas. |  | SPC readiness self assessment tool | Measurement system readiness | Statistical process control readiness in the food industry: Development of a self-assessment tool | 2016 |
| We measure the outcomes of using personas in decision making. |  | SPC readiness self assessment tool | Measurement system readiness | Statistical process control readiness in the food industry: Development of a self-assessment tool | 2016 |
| Our organization has guidelines for the use of personas in decision making. |  | SPC readiness self assessment tool | Measurement system readiness | Statistical process control readiness in the food industry: Development of a self-assessment tool | 2016 |
| Our decision-making about customers is based on data. |  | SPC readiness self assessment tool | Organizational culture readiness | Statistical process control readiness in the food industry: Development of a self-assessment tool | 2016 |
| Everyone participates in persona creation. |  | SPC readiness self assessment tool | Organizational culture readiness | Statistical process control readiness in the food industry: Development of a self-assessment tool | 2016 |
| Persona performance is measured using appropriate metrics (e.g., customer satisfaction, loyalty, sales). |  | SPC readiness self assessment tool | Organizational culture readiness | Statistical process control readiness in the food industry: Development of a self-assessment tool | 2016 |
| Personas are regularly used in team meetings. |  | SPC readiness self assessment tool | Organizational culture readiness | Statistical process control readiness in the food industry: Development of a self-assessment tool | 2016 |
| Top management communicates legitimate reasons for adopting personas. |  | SPC readiness self assessment tool | Urgency to change | Statistical process control readiness in the food industry: Development of a self-assessment tool | 2016 |
| Using personas in our organization can help continuously improve our performance among real customers. |  | SPC readiness self assessment tool | Urgency to change | Statistical process control readiness in the food industry: Development of a self-assessment tool | 2016 |
| Employees are trained in the use of personas. |  | SPC readiness self assessment tool | Employee involvement | Statistical process control readiness in the food industry: Development of a self-assessment tool | 2016 |
| Employees understand the beneﬁts of using personas to the business and themselves. |  | SPC readiness self assessment tool | Employee involvement | Statistical process control readiness in the food industry: Development of a self-assessment tool | 2016 |
| My organization has a culture that accepts the use of personas to make decisions. |  | Learning Analytics Readiness Instrument (LARI) | Culture | The Learning Analytics Readiness Instrument | 2016 |
| We have the ability to store increasingly large volumes of data about our customers. |  | Learning Analytics Readiness Instrument (LARI) | Data Management Expertise | The Learning Analytics Readiness Instrument | 2016 |
| We have professionals with mathematical/statistical experience in manipulating and transforming user data and/or variables in large, complex data sets. |  | Learning Analytics Readiness Instrument (LARI) | Data Analysis Expertise | The Learning Analytics Readiness Instrument | 2016 |
| My institution has professionals who have specialized training in personas. |  | Learning Analytics Readiness Instrument (LARI) | Training | The Learning Analytics Readiness Instrument | 2016 |
| We intend to use personas for strategic decision making (e.g., formulate a customer strategy using personas). |  | Venkatesh and Davis (2000) - TAM2 | Behavioral intention |  |  |
| We intend to use personas for tactical decision making (e.g., plan campaigns using personas). |  |  |  |  |  |
| We intend to use personas for operational decision making (e.g., create content using personas). |  |  |  |  |  |
| I predict that we will be using personas in the short term. |  | Venkatesh and Davis (2000) - TAM2 | Behavioral intention |  |  |
| We have the resources necessary to create personas. |  | Venkatesh et al. (2012) - UTAUT | Facilitating Conditions |  |  |
| We have the knowledge necessary to use personas. |  | Venkatesh et al. (2012) - UTAUT | Facilitating Conditions |  |  |
| Personas are compatible with other technologies and applications we use. |  | Venkatesh et al. (2012) - UTAUT | Facilitating Conditions |  |  |
| We can get help from others when we have difficulties using personas. |  | Venkatesh et al. (2012) - UTAUT | Facilitating Conditions |  |  |
| I personally think personas are valuable for our organization. |  | CONTROL: persona champion or not |  |  |  |
| I believe in personas as a method. |  | CONTROL: persona champion or not |  |  |  |
| We measure the return of investment (ROI) of using personas. |  |  |  |  |  |
| People using the personas for decision making also participated in their creation. |  |  |  |  |  |
|  |  |  |  |  |  |
